# Phylogenomic Investigation of Phospholipid Synthesis in Archaea

**DOI:** 10.1155/2012/630910

**Published:** 2012-12-16

**Authors:** Jonathan Lombard, Purificación López-García, David Moreira

**Affiliations:** Unité d'Ecologie Systématique et Evolution, CNRS UMR 8079, Université Paris-Sud, 91405 Orsay Cedex, France

## Abstract

Archaea have idiosyncratic cell membranes usually based on phospholipids containing glycerol-1-phosphate linked by ether bonds to isoprenoid lateral chains. Since these phospholipids strongly differ from those of bacteria and eukaryotes, the origin of the archaeal membranes (and by extension, of all cellular membranes) was enigmatic and called for accurate evolutionary studies. In this paper we review some recent phylogenomic studies that have revealed a modified mevalonate pathway for the synthesis of isoprenoid precursors in archaea and suggested that this domain uses an atypical pathway of synthesis of fatty acids devoid of any acyl carrier protein, which is essential for this activity in bacteria and eukaryotes. In addition, we show new or updated phylogenetic analyses of enzymes likely responsible for the isoprenoid chain synthesis from their precursors and the phospholipid synthesis from glycerol phosphate, isoprenoids, and polar head groups. These results support that most of these enzymes can be traced back to the last archaeal common ancestor and, in many cases, even to the last common ancestor of all living organisms.

## 1. Introduction

Archaea were identified as an independent domain of life in the late 1970s thanks to the characterization of differences between their ribosomal RNA molecules and those of bacteria and eukaryotes [1]. At that time, it was already known that the so-called “archaeabacteria” held several atypical biochemical characteristics, of which the most remarkable was their unusual membrane phospholipids. Whereas all bacteria and eukaryotes were known to have membranes based on fatty acids linked by ester bonds to glycerol-phosphate, archaea appeared to have phospholipids composed of isoprenoid chains condensed with glycerol-phosphate by ether linkages [2–6]. Moreover, the archaeal glycerol ethers contained *sn*-glycerol-1-phosphate (G1P), whereas bacterial and eukaryotic ones contained *sn*-glycerol-3-phosphate (G3P) (for review, see [7, 8]). These differences, which have been at the center of an intense debate about the nature of the first cell membranes [9], have progressively been shown to be not so sharp: ether-linked lipids are common both in eukaryotes (up to 25% of the total lipids in certain animal cells, [10]) and in several thermophilic bacteria [11–13]; fatty acid phospholipids have been found in diverse archaea [14]; conversely, isoprenoids are known to be universal although they are synthesized by non-homologous pathways in the three domains of life, [15]; even the stereochemistry of the glycerol phosphate has some exceptions, as shown by the recent discovery of archaeal-like *sn*-glycerol-1-phosphate specific lipids in some bacteria [16] and in eukaryotic endosomes [17].

Today, thanks to the significant accumulation of complete genome sequence data for a wide variety of species, it is possible to study the major pathways of phospholipid synthesis and their exceptions by looking at the presence or absence of the corresponding genes in the different genomes. In addition, these data allows reconstructing the evolutionary history of each gene by combining comparative genomics and phylogenetics, namely, by using a phylogenomic approach. In this paper, we summarize the results concerning the phylogenomic studies of the biosynthesis pathways of archaeal phospholipid components and provide some new evolutionary data about the enzymes reviewed in Koga and Morii [18] and Matsumi et al. [19] as likely responsible for the assembly of archaeal lipids from these building blocks.

## 2. Archaea Possess an Atypical Pathway of Biosynthesis of Isoprenoid Precursors

**Figure 1:**
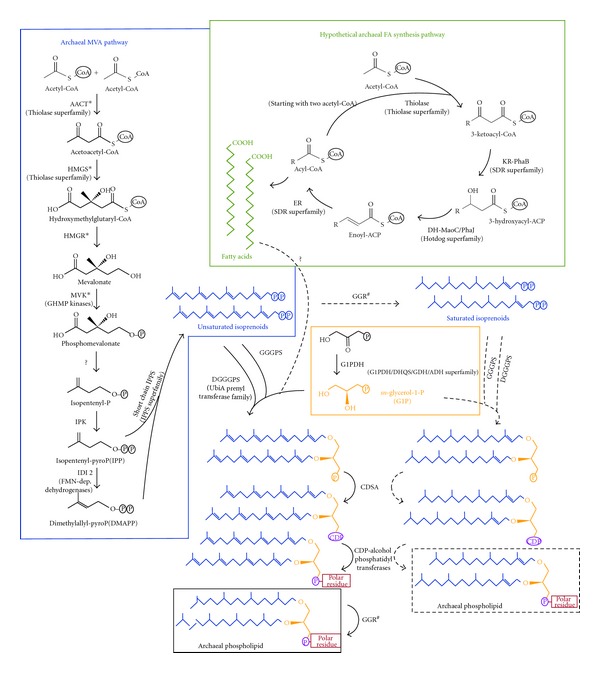
Biosynthesis pathways of phospholipid components in archaea. Abbreviations for the archaeal mevalonate (MVA) pathway: AACT, acetoacetyl-CoA thiolase; HMGS, 3-hydroxy-3-methylglutaryl-CoA synthase; HMGR, 3-hydroxy-3-methylglutaryl-CoA reductase; MVK, mevalonate kinase; IPK, isopentenyl phosphate kinase; IDI2, isopentenyl diphosphate isomerase type II. GHMP, galactokinase-homoserine kinase-mevalonate kinase-phosphomevalonate kinase; IPPS, isoprenyl diphosphate synthases (asterisks indicate enzymes shared with the eukaryotic MVA pathway). Abbreviations for the hypothetical archaeal fatty acid (FA) synthesis pathway: ACC, acetyl-CoA carboxylase; PCC, propionyl-CoA carboxylase; KR-PhaB, beta-ketoacyl reductase; DH-MaoC/PhaJ, beta-hydroxyacyl dehydratase; ER, enoyl reductase; SDR, short-chain dehydrogenases/reductases. Abbreviations for the *sn*-glycerol-1-phosphate synthesis pathway: G1PDH, glycerol-1-phosphate dehydrogenase; DHQS, 3-dehydroquinate synthase; GDH, glycerol dehydrogenase; ADH, alcohol dehydrogenase. Abbreviations for the phospholipid assembly pathway: GGGPS, (S)-3-O-geranylgeranylglyceryl phosphate synthase; DGGGPS, (S)-2,3-di-O-geranylgeranylglyceryl phosphate synthase; GGR, geranylgeranyl reductase; CDSA, CDP diglyceride synthetase. (^#^) points to the ability of GGRs to reduce isoprenoids at different steps in the biosynthesis pathway. Names between parentheses indicate the family or superfamily to which belong the archaeal enzymes postulated to carry out particular functions on the basis of phylogenomic analyses.

Isoprenoids are chains of isoprene units and their derivatives and are found ubiquitously in all living beings. They are involved in very diverse functions, such as photosynthetic pigments, hormones, quinones acting in electron transport chains, plant defense compounds, and so forth [15, 20]. In archaea, isoprenoids also make up the hydrophobic lateral chains of phospholipids [7]. Isoprenoid biosynthesis requires two isoprene activated precursors, called isopentenyl pyrophosphate (IPP) and dimethylallyl diphosphate (DMAPP), which act as building parts. The first metabolic pathway responsible for the biosynthesis of isoprenoid precursors was discovered in yeasts and animals in the 1950s and named mevalonate (MVA) pathway in reference to its first committed precursor. Since its first description, the MVA pathway was thought to synthesize the isoprenoid precursors in all organisms [20], but closer scrutiny showed its absence in most bacteria [21]. An elegant series of multidisciplinary approaches allowed in the late 1990s and early 2000s describing an independent and non-homologous pathway in bacteria, the methylerythritol phosphate (MEP) pathway (see [22] for review). The MEP pathway was found to be widespread in bacteria and plastid-bearing eukaryotes, whereas the MVA pathway was assumed to operate in archaea and eukaryotes [15, 23]. In the early 2000s, archaeal isoprenoids were thought to be synthesized through the MVA pathway because the ^14^C-labelled intermediates of this pathway were shown to be incorporated into the archaeal phospholipids [24]. However, only two of the enzymes of the eukaryotic pathway have been described in archaea [25, 26]. Moreover, the search of MVA pathway enzyme sequences in archaeal genomes revealed that from the seven enzymes acting on this pathway, most archaeal species lack the last three of them: phosphomevalonate kinase (PMK), mevalonate diphosphate decarboxylase (MDC), and isopentenyl diphosphate isomerase (IDI1), involved in the conversion of phosphomevalonate into IPP and DMAPP [27]. Attempts to characterize these missing archaeal enzymes revealed two new enzymes: the isopentenyl phosphate kinase (IPK) and an alternative isopentenyl diphosphate isomerase (IDI2) [28–30]. Although the decarboxylation reaction required to synthesize isopentenyl phosphate from phosphomevalonate has not been described yet, an alternative final MVA pathway involving IPK and IDI2 was then proposed to exist in archaea (Figure 1, [29]).

Taking advantage of the recent accumulation of genomic data, we carried out a thorough phylogenomic analysis of the pathways of isoprenoid precursor synthesis [31]. Concerning archaea, our presence-and-absence analysis extended the presence of the proposed archaeal alternative pathway to a wide archaeal diversity. The main exception to these observations was *Nanoarchaeum equitans*, which explains the dependence of this organism to obtain its lipids from its crenarchaeal host, *Ignicoccus hospitalis* [32]. Phylogenetic reconstructions of the MVA pathway enzymes present in archaea systematically revealed a clade independent from the other domains of life, representative of the archaeal diversity and generally respecting the main archaeal taxonomic groups [31]. These results are consistent with the presence of a distinctive archaeal MVA pathway in the last archaeal common ancestor (LACA) and support that different isoprenoid precursor biosynthesis pathways are characteristic of each domain of life: the classical MVA pathway in eukaryotes, the alternative MVA pathway in archaea, and the MEP pathway in bacteria.

The origin of the archaeal MVA pathway appears to be composite. The enzymes shared with the classical eukaryotic MVA pathway were also ancestral in eukaryotes and bacteria and, therefore, they can be inferred to have been present in the last common ancestor of all living organisms (the cenancestor), from which the archaeal lineage would have inherited them. The last enzymes of the eukaryotic MVA pathway were most likely present in the respective ancestors of eukaryotes and bacteria, but their distribution in archaea is scarce and complex: homologues of MDC can be detected in Haloarchaea and Thermoplasmatales and some IDI1 genes have been identified in Haloarchaea and Thaumarchaeota, although they most likely reflect recent horizontal gene transfer (HGT) events from bacterial donors, as deduced from their position well nested among bacterial sequences in phylogenetic analyses [23, 31]. In contrast, the class Sulfolobales contains PMK and MDC homologues which robustly branch in an intermediate position between the eukaryotic and the bacterial sequences, suggesting that these sequences could be ancestral versions that would have been lost in the rest of archaea [31]. If this is the case, a eukaryotic-like pathway (except for the IDI function, which remains ambiguous) can be proposed to have existed in the cenancestor. This pathway would have been replaced by the MEP pathway in the bacterial lineage, whereas in archaea only the last steps were replaced by non-homologous enzymes. We do not know the evolutionary forces that drove these changes; however, the mevalonate kinase (MVK), PMK, and MDC belong to a large family of kinases, namely the GHMP kinases [33], which is characterized by a high structural and mechanistic conservation that contrasts with the large range of substrates that they can use [34]. If we assume that ancestors of these enzymes were not very specific, this could have allowed some tolerance to recruitment of evolutionary unrelated enzymes not only in archaea but also in some eukaryotes that have replaced their ancestral PMK by a non-homologous one [35, 36]. In agreement with this idea, the archaeal IPK itself appears to be able to use different substrates [37], which could have made the replacement easier.

## 3. Early Evolution of Isoprenoid Chain Synthesis

Once the isoprenoid precursors, IPP and DMAPP, have been synthesized, they still have to be assembled to make isoprenoid chains. The prenyltransferases responsible for this function are the isoprenyl diphosphate synthases (IPPS). Since many more complete genome sequences are now available than at the time of previous IPPS phylogenetic analyses, we present here an updated survey of the evolution of these enzymes. Although many different enzymes are required to synthesize the wide diversity of isoprenoids, the first steps are widely shared among the three domains of life [38]. They consist of the progressive addition of IPP units (5 carbons each) to an elongating allyl polyisoprenoid diphosphate molecule. Starting with DMAPP (5 carbons), consecutive condensation reactions produce geranylgeranyl diphosphate (GPP, 10 carbons), farnesyl diphosphate (FPP, 15 carbons), geranylgeranyl diphosphate (GGPP, 20 carbons), and so forth. Different IPPSs are characterized by the allylic substrate that they accept (DMAPP, GPP, FPP,…) and by the stereochemistry of the double bonds, but the main characteristic used to classify them is the size of the products that they synthesize: they can be short-chain IPPS (~up to 25 carbons) or long-chain IPPS (>25 carbons). All IPPS are homologous and share a common reaction mechanism. Short-chain IPPS mainly differ in the size of their substrate-binding hydrophobic pocket (the smaller the pocket, the shorter the final product, [38]), but point mutations have been shown to importantly impact the size of the pocket and, thus, of their final products [39, 40]. Description of equivalent natural mutations have been reported in archaea [41], which argues against the possibility of inferring the precise product of a given IPPS only based on its phylogenetic position [41, 42].

The first published IPPS phylogenetic trees [42] only used 13 sequences (of which 12 were short-chain enzymes) and supported a split between prokaryotic and eukaryotic enzymes. At that time, archaeal IPPS were assumed to be more ancient because they were known to provide isoprenoids for several pathways while bacteria and eukaryotes were thought to have more specialized enzymes. Later phylogenetic analyses with more sequences showed two groups corresponding to the functional split between short and long-chain enzymes [41]. This was expected since the long-chain IPPS use supplementary proteins to stabilize the hydrophobic substrate [38], which would logically impact the protein structure and therefore trigger the large divergence between the short- and the long-chain enzymes. In that work, the short-chain enzymes formed three groups according to the three domains of life, which could be considered as evidence for the presence of one short-chain enzyme in the cenancestor and its subsequent inheritance in modern lineages, including archaea. Furthermore, in that phylogeny archaea did not branch as a basal lineage, disproving their previously assumed ancient character. In addition, enzymes with different product specificities branched mixed all over the tree, supporting the product plasticity of the ancestral short-chain enzyme and the subsequent evolution of particular specificities according to biological requirements in modern organisms [41]. Archaeal long-chain IPPS had not been detected in that study, but some of them were incorporated in phylogenetic studies some years later [23].

**Figure 2:**
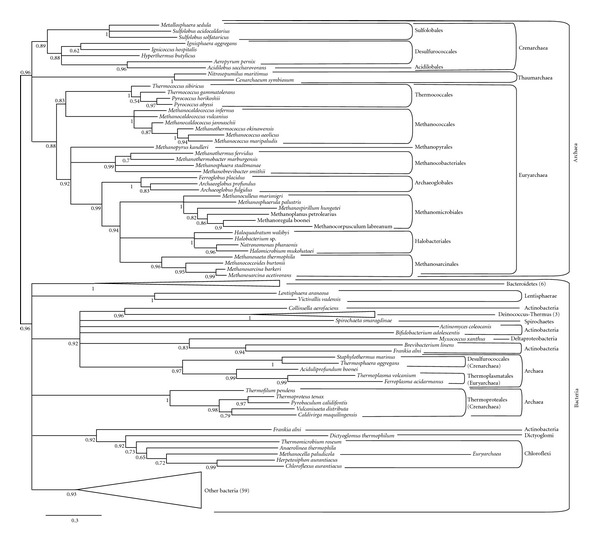
Short-chain IPPS phylogenetic tree reconstructed using 136 representative sequences and 244 conserved sites. Multifurcations correspond to branches with support values <0.50. Triangles correspond to well supported-bacterial clades (numbers in parentheses correspond to the number of sequences included in these clades). For the complete phylogeny, see Supplementary Figure 1.

We have searched for homologues of IPPS in a representative set of 348 complete genomes from the three domains of life (including 88 archaea). A preliminary phylogenetic analysis showed that, in agreement with previous reports [23], the resulting sequences split into two clades mainly related to the short- or long-chain product specificity (data not shown). To avoid the phylogenetic artifacts that can be introduced by the high sequence divergence between short- and long-chain enzymes, we carried out independent phylogenetic analyses for each one of the two paralogues, to which we will refer as short- or long-chain IPPS with regard to dominant functions of the characterized enzymes. However, as previously mentioned [39–41], substrate specificity exchange between short and long substrates appears to be relatively common, so these partial trees must be acknowledged mainly as phylogenetic groups related to the most widespread biochemical function, but do not definitely determine the substrate specificity of all their sequences, which can only be established by biochemical studies. In preliminary short-chain IPPS phylogenies, long branches at the base of several eukaryotic paralogues evidenced for the high divergence of these sequences. Since we were here mainly interested in the archaeal sequences, we removed the divergent eukaryotic sequences from our analyses.  Figure 2 and Supplementary Figure 1 (see Supplementary Material available online at doi:10.1155/2012/630910) present the phylogeny of some representative prokaryotic short-chain enzyme sequences. Most archaeal sequences branch together in a monophyletic group largely congruent with the main archaeal phyla and orders, which suggests that this enzyme was present in LACA and was vertically inherited in most archaeal lineages. Most bacterial sequences also cluster together according to the main bacterial taxonomic groups, suggesting the ancestral presence of this enzyme in the last bacterial common ancestor and, given its presence also in LACA, most likely also in the cenancestor. However, some recent HGTs can also be pointed out from this phylogeny. Concerning archaea, Thermoproteales, some Desulfurococcales, some Thermoplasmatales, *Methanocella paludicola* and *Aciduliprofundum boonei* branch within the bacterial group, so several HGTs can be invoked to explain this pattern, although in most cases the support is weak and does not allow confidently determining the identity of the bacterial donors (Figure 2).

**Figure 3:**
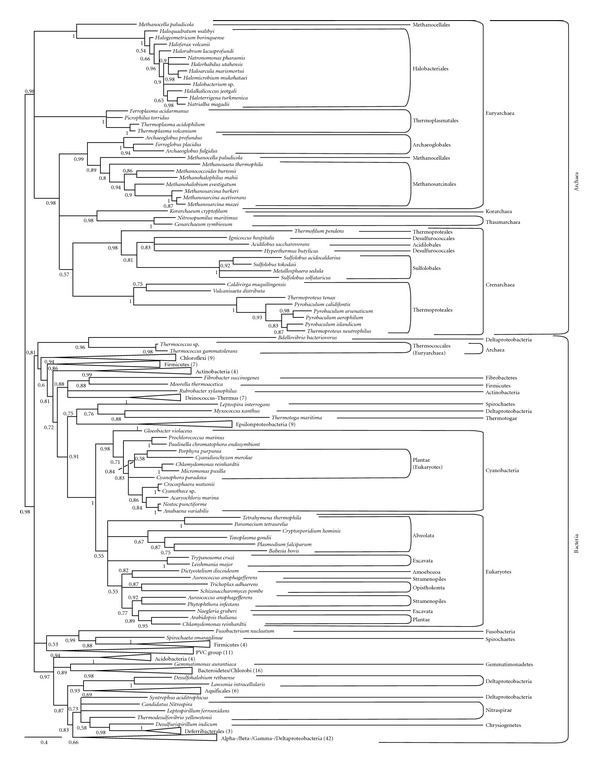
Long-chain IPPS phylogenetic tree reconstructed using 218 representative sequences and 241 conserved sites. Multifurcations correspond to branches with support values <0.50. Triangles correspond to well-supported clades outside Archaea (numbers in parentheses correspond to the number of sequences included in these clades). For the complete phylogeny, see Supplementary Figure 2.

As in the case of the short-chain enzymes, most archaeal sequences also group together in our long-chain IPPS phylogenies and, despite the weakly supported paraphyly of the euryarchaeotal sequences, the main archaeal taxonomic groups are observed (Figure 3 and Supplementary Figure 2). Bacterial sequences also group together according to main taxa, supporting that the respective common ancestors of bacteria and archaea, and thus likely also the cenancestor, had a long-chain IPPS enzyme that was inherited in modern organisms. Contrary to short-chain IPPS, eukaryotic long-chain IPPS are less divergent, so they were conserved in our analyses. Surprisingly, all the eukaryotic sequences, including those from plastid-lacking eukaryotes, branch together as the sister group of cyanobacteria, except for some algae that branch within the cyanobacterial group. In addition, some other more recent HGTs are observed, especially in the archaeal *Thermococcus* genus, which branches close to a very divergent sequence from the delta-proteobacterium *Bdellovibrio bacteriovorus*.

Altogether, these results support that in spite of some recent HGTs from bacteria to archaea, most archaea have homologues of both the short- and the long-chain IPPS that were inherited from LACA and that, most likely, were already present in the cenancestor.

## 4. Archaea May Have an ACP-Independent Fatty Acid Biosynthesis Pathway

In bacteria and eukaryotes, fatty acids are components of membrane phospholipids, energy-storage molecules, substrates for post-translational modifications of proteins, secondary metabolites, and components of coenzymes and messenger compounds. Despite the general assumption that archaeal lipid metabolism is based on isoprenoids, a variety of experimental approaches have shown that fatty acids (FA) also exist in these organisms. Archaea show variable concentrations of free FA or their derivatives [4, 24, 43–45], which has stimulated some attempts to biochemically characterize an archaeal FA synthase [46, 47]. Archaeal FA can participate in protein structure [48–50] and acylation [47] but they have also been found as components of membrane phospholipids in very diverse euryarchaeotes [14]. Therefore, it is not surprising that homologues of several of the enzymes involved FA biosynthesis in bacteria had been detected in archaeal genomes [8, 51]. In bacteria, FA biosynthesis occurs through multiple condensations of acyl groups (malonyl-CoA) in a series of cyclical steps. The building blocks necessary to the activity of the FA synthases are provided by several reactions. First, the acetyl-CoA carboxylase (ACC) converts acetyl-coenzyme A (CoA) into malonyl-CoA. Second, the peptide cofactor acyl carrier protein (ACP), which is required to channel the elongating intermediates among the FA synthase enzymes, has to be activated through the addition of a phosphopantetheine group from CoA to the apo-ACP by an ACP synthase. Finally, the malonyl-CoA: ACP transacylase (MCAT) charges the malonyl-CoA to holo-ACP, resulting in malonyl-ACP.

Since no archaeal FA synthase system has been described in detail yet, we recently studied the evolution of the archaeal homologues of the bacterial genes involved in FA synthesis. First, many archaea possess ACC homologues that are closely related in phylogenetic analyses, suggesting their possible monophyletic origin and, consequently, the presence of this enzyme in LACA [52, 53]. Second, only a few unrelated archaeal species have ACP and the ACP-processing machinery (ACP synthase and MCAT) and phylogenetic analysis suggests that they acquired them by HGTs from bacteria. As a result, ACP and its related enzymes are missing in most archaea and appear not to have been present in LACA [54]. For the rest of enzymes involved in the cyclic steps of FA synthesis (see Figure 1), phylogenetic analyses support that the archaeal sequences are more closely related to bacterial enzymes that are active on substrates linked to CoA than to enzymes that use substrates linked to ACP. Taking this into account, we have proposed that the ACP processing system has specifically evolved in the bacterial lineage and that archaea carry out FA synthesis using a likely ancient ACP-independent pathway [54]. Although ACC exists in archaea, the hypothetical archaeal FA synthesis pathway probably involves two acetyl-CoA instead of using malonyl and acetyl thioesters as in bacteria, since the thiolases that carry out this function are known to be decarboxylative in bacteria but non-decarboxylative in archaea [55].

Interestingly, it has been unclear for a long time if either the bacterial FA synthesis pathway specifically recognizes each acyl-ACP intermediate or if its enzymes randomly fix these intermediates, modifying the correct ones and releasing the inappropriate ones. Recent work has shown that ACP carries out its channeling function by adopting unique conformations for each enzyme of the FA elongation cycle [56]. Yet, it can be reasonably assumed that a putative ACP-independent mechanism would rather use random interactions between intermediate metabolites and enzymes, which might be assumed to be less efficient than the bacterial ACP-mediated system. Although this remains speculative and needs biochemical confirmation, the high efficiency of the ACP-mediated machinery may explain the preeminence of FA in bacterial membranes, whereas archaea would have opted by the alternative ancestral mechanism of synthesis of lateral chains for membrane phospholipids, namely, the isoprenoids [31], relegating FA to different cellular functions and, only to a small extent, to membrane synthesis [54].

## 5. Linking Glycerol Phosphate with the Lateral Chains

Although archaeal phospholipids were already known to use G1P instead of G3P as bacteria and eukaryotes do, the recent characterization and sequencing of the enzyme responsible for its synthesis in archaea (G1P dehydrogenase [57, 58]) was astonishing because this enzyme appeared to be totally unrelated to the canonical G3P dehydrogenase [59]. Since then, very little exceptions to this clear-cut distinction between archaea and bacteria have been described (the most remarkable is probably the description of an archaeal-like G1P dehydrogenase in the bacterium *Bacillus subtilis*, [60]), but the fact that the two dehydrogenases belong to large enzymatic superfamilies from which they were recruited has provided a model for the independent origin of these dehydrogenases from likely ancient promiscuous enzymes [8].

Geranylgeranylglyceryl diphosphate and di-O-geranylgeranylglyceryl phosphate synthases (GGGPS and DGGGPS, resp.) are the enzymes that subsequently link isoprenoids to glycerol phosphate in archaea [18]. Boucher et al. [23] found that GGGPS are widespread in archaea whereas, among bacteria, it was found only in Bacillales and in one Bacteroidetes species. They also described a very divergent homologue in halophilic archaea and in *Archaeoglobus*. Despite their divergence, these enzymes appear to carry out the same function [61]. Using an updated genome sequence database, we also retrieve the very divergent GGGPS in Methanomicrobiales, confirm that GGGPS is present in Bacillales, and extend this observation to a surprising diversity of Bacteroidetes (Supplementary Figure 3. This suggests that GGGPS plays an important role in these two bacterial groups and, indeed, it has recently been shown to participate in the synthesis of archaeal-type lipids of unknown function in these bacteria [16]. In conclusion, at least one GGGPS gene appears to have been present in LACA, whereas the divergent copy of this gene probably emerged later in a more recent group of euryarchaeota. GGGPS were probably independently transferred to the respective ancestors of Bacillales and Bacteroidetes.

Hemmi et al. [62] carried out the first phylogenetic analysis of the DGGGPS sequences and their superfamily, the UbiA prenyltransferase family (17 sequences branching in 6 different groups). Using all sequences available at present, we retrieve similar but much more diversified groups (Supplementary Figure 4. Archaeal sequences cluster together in a monophyletic assemblage, suggesting common ancestry, although crenarchaeota are paraphyletic probably as a result of reconstruction artifacts. A number of bacteroidetes sequences branch among the crenarchaeota, reflecting an HGT event to an ancestor of this bacterial group. In addition several bacterial phyla (Chlorobi, Cyanobacteria, Chloroflexi, Planctomycetales, and some proteobacterial species) possess UbiA-related homologues. However, in several of these bacterial species, these enzymes are involved in photosynthesis (they take part in the synthesis of respiratory quinones, hemes, chrolophylls, and vitamin E [62]) and it is difficult to determine if the widespread distribution in bacteria is due to the ancestral presence of a homologue of this enzyme in the last common bacterial ancestor or to several recent HGTs to these bacterial groups from archaeal donors.

The search of homologues of the bacterial glycerol phosphate acyl transferases PlsX, PlsY, PlsB, and PlsC, which carry out the addition of fatty acids to glycerol phosphate [63, 64], in the available archaeal genome sequences allowed retrieving very few homologues, all of them most likely acquired by HGT from bacterial donors (not shown).

## 6. Linking the Polar Head Groups

**Figure 4:**
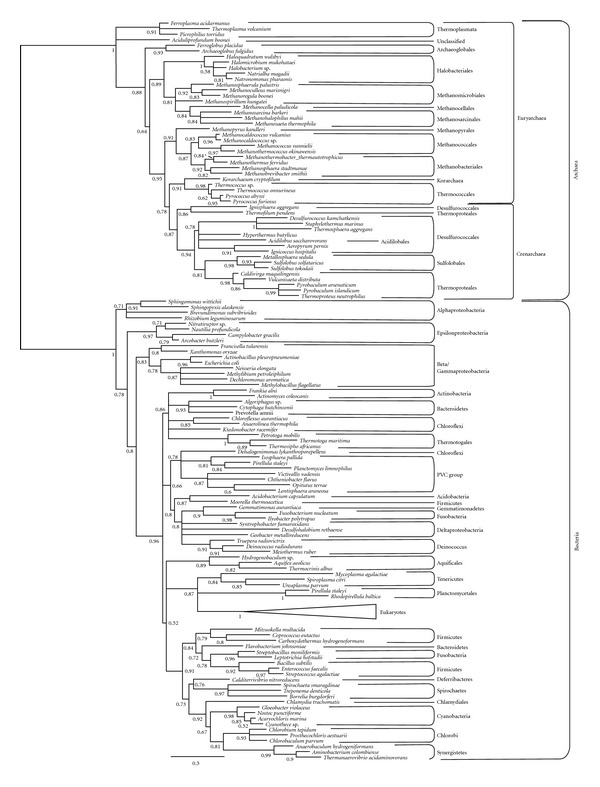
CdsA phylogenetic tree reconstructed using 133 representative sequences and 87 conserved sites. Branches with support values <0.50 have been collapsed. For the complete phylogeny, see Supplementary Figure 8.

Once the two hydrocarbon chains are linked to the phospholipid backbone, two additional steps are required to add the polar head group (Figure 1). First, an enzyme replaces the phosphate linked to the glycerol moiety by a CDP group; then, this CDP is replaced by the final polar head group, which can be very diverse (glycerol, myo-inositol, serine, and so forth) [18]. The first step has been biochemically described in archaea but the enzyme that carries out this function remains unknown [65]. The bacterial counterpart is the CDP diglyceride synthetase (CdsA) [66, 67]. We looked for archaeal homologues of the bacterial enzyme and retrieved several sequences that we used as queries for exhaustive searches in archaeal genomes. This allowed us to observe that this enzyme is widespread both in bacteria and archaea. Our phylogenetic trees show two clades that correspond to archaea and bacteria and the phylogenetic relationships within each of them are congruent with the main accepted taxonomic groups (Figure 4). This supports the vertical inheritance of this gene from the cenancestor and, consequently, its presence in LACA. However, to our knowledge no archaeal CdsA has been biochemically characterized so far, which would be required to confirm that these archaeal homologues are responsible for the biochemical activity described by Morii et al. [65].

Two enzymes involved in the second step of the attachment of polar head groups have been characterized in archaea [68, 69]. These enzymes belong to the large family of the CDP alcohol phosphatidyltransferases and have homologues in bacteria [70]. The bacterial members of this enzyme family are known to have different specificities in order to add different polar head groups on phospholipids. A previous phylogenomic survey carried out by Daiyasu et al. [70] showed that the different sequences group in phylogenetic trees according to their predicted substrates. In addition, the groups of bacterial sequences were in agreement with the main bacterial taxonomic groups. Our analysis with a larger taxonomic sample confirms that homologues of these genes are widespread in archaea. All sequences group into three main categories (data not shown), one related to the addition of serine as a polar head group [68], another related to myo-inositol-phosphate transfer [69] (and maybe also glycerol in archaea, according to classification in [70]), and the last one using glycerol. Archaea are found in the first two groups but not in the last one. In order to avoid artifacts due to extreme sequence divergence, we carried out phylogenies for each of the two groups containing archaeal representatives. In the first phylogeny, archaeal sequences predicted to use serine [68] as a substrate are limited to Euryarchaeota (Supplementary Figure 5). This tree shows a poorly supported group of slow evolving bacteria together with several divergent bacterial, eukaryotic and archaeal (*Methanococcoides burtonii* and haloarchaea) sequences. Such mixed distribution dominated by strong sequence divergence and HGTs contrasts with the rest of the phylogeny, which is congruent with the main accepted taxonomic groups. Especially, one monophyletic group of euryarchaeota can be pointed out, supporting that this enzyme probably existed at least in the last euryarchaeotal common ancestor. In the second tree, a larger group of archaeal enzymes, predicted to use glycerol phosphate or myo-inositol-phosphate as substrates [69, 70], cluster in our analysis with bacterial enzymes that use myo-inositol, but these bacterial sequences are scarce and appear to have been acquired from archaea by HGT (Supplementary Figure 6). The phylogeny of archaeal sequences supports the monophyly of Crenarchaeota and a patchy distribution of several paralogues in Euryarchaeota, which makes difficult the analysis of the evolution of these enzymes in archaea without incorporating supplementary biochemical information. At any rate, the wide distribution of this enzyme family in archaea strongly suggests that at least one representative of these enzymes was already present in LACA. Whereas it was conserved in Crenarchaeota, it was subjected to several duplication and neofunctionalization events in Euryarchaeota, which would explain the complex distribution pattern observed in euryarchaeotal species.

## 7. Saturation of Isoprenoid Chains

So far, we have described the main synthesis and link mechanisms of archaeal phospholipid components, but a wide diversity of phospholipids actually exists in archaea [71]. A substantial characteristic of archaeal membranes is their saturation rate, since double bonds have a prominent influence on membrane stability [72]. Most archaea contain saturated phospholipids and the geranylgeranyl reductase (GGR), the enzyme responsible for the reduction of isoprenoid chains, has been recently described in the euryarchaeota *Thermoplasma acidophilum* and *Archaeoglobus fulgidus* and the crenarchaeote *Sulfolobus acidocaldarius* [73–75]. These studies show that archaeal GGR are able to use geranylgeranyl chains either isolated or attached to phospholipids as substrates. When GGPP is used as a substrate, all bonds but the one in position C2 are reduced, allowing the incorporation in phospholipids, but isoprenoids chains that are already attached to glycerol phosphate can have all their double bonds reduced by this enzyme. As a result, isoprenoid reduction can happen at different steps of the phospholipid biosynthesis pathway (Figure 1).

Archaeal GGRs are homologous to previously reported cyanobacterial and plant GGRs involved in chlorophyll synthesis [76, 77] and many different GGR paralogues have been identified in archaeal genomes that could carry out independent isoprenoid chain reductions [19]. Our phylogeny of GGRs confirms that GGRs are widespread among crenarchaeota and that at least two paralogues exist in a wide diversity of euryarchaeota, although some archaeal groups also bear supplementary GGR genes (Supplementary Figure 7). This supports the ancestral presence of at least one GGR gene in archaea, followed by a complex history of duplications and HGTs. GGRs are also present in many bacterial genomes, but several HGTs can be observed, probably related to the function of this gene in photosynthesis, making uncertain the primary origin of this gene in bacteria. Among eukaryotes, only sequences from plastid-bearing organisms were detected and these sequences clearly branched within one group of cyanobacterial sequences, thus supporting the plastidial origin of these genes in eukaryotes.

## 8. Discussion

In contrast with the rather impressive knowledge about the biochemistry and biosynthesis of bacterial cell membranes, many aspects of their archaeal counterparts remain to be elucidated. Even the synthesis of the most canonical archaeal membrane lipids, the phospholipids based on isoprenoid lateral chains, still has some unresolved points, such as the identity of the enzyme carrying out the phosphomevalonate decarboxylase activity necessary for the final steps of the synthesis of isoprenoid precursors. Moreover, it is becoming clear that archaeal membranes incorporate components that were supposed to be restricted to the other two domains of life, bacteria and eukaryotes. This is notably the case of fatty acids that, despite being relatively widespread in membrane phospholipids of euryarchaeotal species [14], are synthesized by a pathway that, most likely, has important differences with the bacterial counterpart [54].

Comparative genomics and phylogenomics provide a powerful way to address these questions, in particular by the detection of potential candidates to carry out missing enzymatic functions thanks to similarities with bacterial and eukaryotic enzymes. This has allowed us to propose, for example, the existence of an ACP-independent pathway of fatty acid synthesis in archaea [54] and allows us to propose here that archaeal CdsA homologues could carry the same function as in bacteria. This approach has also provided evidence supporting that lipid membranes were already evolved long ago, at the time of the cenancestor [9]. Thus, rather than radical inventions of new phospholipid biochemistries, bacteria and archaea appear to have specialized their cell membranes by tuning the relative importance of the different components, with isoprenoids becoming dominant in archaea and fatty acids in bacteria. Nevertheless, these bioinformatic approaches have limitations and biochemical investigation remains crucial to characterize the different missing activities (the uncharacterized MVA pathway enzymes in archaea, the hypothetical ACP-independent FA synthesis pathway, the characterization of the archaeal CdsA, and a larger diversity of CDP alcohol phosphatidyltransferases) in order to complete the puzzle of archaeal membrane synthesis.

## 9. Material and Methods

Sequence seeds for similarity searches were retrieved from the KEGG database (http://www.genome.jp/kegg/). Searches were carried out with BLASTp [78] against a list of completely sequenced genomes available in GenBank (Supplementary Table 1). The resulting sequences were aligned with by default parameters with Muscle 3.6 [79]. Redundant and partial sequences were removed and ambiguously aligned regions were discarded prior to phylogenetic analyses using the NET program from the MUST package [80]. Phylogenetic trees were reconstructed with the approximately maximum likelihood approach with FastTree 2.1.3 [81].

## Supplementary Material

The supplementary material contains a list of the completely sequenced genomes used in this study and the detailed phylogenies of the short and long chain IPPS, the GGGPS, the DGGGPS, the CDP alcohol phosphatidyltransferases probably related to serine transfer, the CDP alcohol phosphatidyltransferases probably related to myo-inositol transfer and the GGR.
