# Supplementary material for: Phylogenomic Investigation of Phospholipid Synthesis in Archaea
Source: Archaea. 2012 Dec 16;2012:630910. doi: 10.1155/2012/630910 (PMC3533463; doi:10.1155/2012/630910)
Supplement: Supplementary file 1 — The supplementary material contains a list of the completely sequenced genomes used in this study and the detailed phylogenies of the short and long chain IPPS, the GGGPS, the DGGGPS, the CDP alcohol phosphatidyltransferases probably related to serine transfer, the CDP alcohol phosphatidyltransferases probably related to myo-inositol transfer and the GGR. [file 630910.f1.pdf]

Supplementary Material

Phylogenomic Investigation of Phospholipid Synthesis in Archaea

Jonathan Lombard, Purificación López-García, and David Moreira

SUPPLEMENTARY TABLE 1: Species used in similarity searches

SUPPLEMENTARY FIGURE 1: Complete short-chain IPPS phylogeny.

SUPPLEMENTARY FIGURE 2: Complete long-chain IPPS phylogeny.

SUPPLEMENTARY FIGURE 3: GGGPS phylogeny.

SUPPLEMENTARY FIGURE 4: DGGGPS phylogeny.

SUPPLEMENTARY FIGURE 5: Phylogenetic tree of CDP alcohol phosphatidyltransferases probably related to serine transfer.

SUPPLEMENTARY FIGURE 6: Phylogenetic tree of CDP alcohol phosphatidyltransferases probably related to *myo*-inositol transfer.

SUPPLEMENTARY FIGURE 7: Phylogenetic tree of geranylgeranyl reductases (GGR).

SUPPLEMENTARY FIGURE 8: Phylogenetic tree of CDP diglyceryde synthases (CdsA).

SUPPLEMENTARY TABLE 1: Species used in similarity searches

**Bacteria**	*Acaryochloris marina MBIC11017*
	Acholeplasma laidlawii PG-8A
	Acidimicrobium ferrooxidans DSM 10331
	Acidithiobacillus ferrooxidans ATCC 53993
	Acidobacterium capsulatum ATCC 51196
	Acidovorax citrulli AAC00-1
	Acinetobacter johnsonii SH046
	Actinobacillus pleuropneumoniae
	Actinomyces coleocanis DSM 15436
	Aeromonas hydrophila
	Akkermansia muciniphila ATCC BAA-835
	Algoriphagus sp. PR1
	Alicyclobacillus acidocaldarius
	Alkaliphilus metalliredigens
	Alteromonas macleodii ATCC 27126
	Aminobacterium colombiense DSM 12261
	Aminomonas paucivorans
	Anabaena variabilis
	Anaerobaculum hydrogeniformans
	Anaerolinea thermophila
	Anaplasma marginale str. Florida
	Aquifex aeolicus VF5
	Arcobacter butzleri RM4018
	Arthrospira maxima
	Atopobium parvulum DSM 20469
	Bacillus subtilis BSn5
	Bacteroides uniformis ATCC 8492
	Bdellovibrio bacteriovorus HD100
	Bifidobacterium adolescentis
	Blastopirellula marina DSM 3645
	Bordetella bronchiseptica RB50
	Borrelia burgdorferi 118a
	Brachyspira murdochii DSM 12563
	Brevibacterium linens BL2
	Brevundimonas subvibrioides ATCC 15264
	Burkholderia cenocepacia HI2424
	Calditerrivibrio nitroreducens
	Caminibacter mediatlanticus TB-2
	Campylobacter gracilis RM3268
	Candidatus Koribacter versatilis Ellin345
	Candidatus Nitrospira defluvii
	Candidatus Phytoplasma australiense
	Candidatus Solibacter usitatus Ellin6076
	Carboxydothermus hydrogenoformans
	Carnobacterium sp. AT7
	Caulobacter crescentus CB15
	Chlamydia trachomatis 434/Bu
	Chlamydophila pneumoniae AR39
	Chlorobaculum parvum
	Chlorobium limicola
	Chlorobium luteolum DSM 273
	Chlorobium tepidum TLS
	Chloroflexus aurantiacus
	Chloroherpeton thalassium ATCC 35110
	Chthoniobacter flavus Ellin428
	Collinsella aerofaciens
	Coprococcus eutactus
	Coraliomargarita akajimensis DSM 45221
	Crocosphaera watsonii
	Cyanobium sp.

Cyanothece sp. ATCC 51142
Cytophaga hutchinsonii ATCC 33406
Dechloromonas aromatica
Deferribacter desulfuricans
Dehalococcoides ethenogenes 195
Dehalogenimonas lykanthroporepellens BL-DC-9
Deinococcus geothermalis
Deinococcus radiodurans
Denitrovibrio acetiphilus
Desulfarculus baarsii DSM 2075
Desulfatibacillum alkenivorans AK-01
Desulfococcus oleovorans Hxd3
Desulfohalobium retbaense DSM 5692
Desulfurispirillum indicum
Dethiosulfovibrio peptidovorans DSM 11002
Dictyoglomus thermophilum
Dictyoglomus turgidum DSM 6724
Dokdonia donghaensis MED134
Elusimicrobium minutum
Enterobacter sakazakii ATCC BAA-894
Enterococcus faecalis
Escherichia coli O157:H7 str. EC4024
Eubacterium dolichum
Fervidobacterium nodosum Rt17-B1
Fibrobacter succinogenes
Flavobacterium johnsoniae
Francisella tularensis
Frankia alni ACN14a
Fusobacterium nucleatum subsp. nucleatum ATCC 23726
Gallionella capsiferriformans ES-2
Gemmata obscuriglobus UQM 2246
Gemmatimonas aurantiaca T-27
Geobacter metallireducens GS-15
Gloeobacter violaceus PCC 7421
Halomonas elongata DSM 2581
Helicobacter pylori B38
Heliobacterium modesticaldum
Herpetosiphon aurantiacus
Hydrogenivirga sp. 128-5-R1-1
Hydrogenobaculum sp. Y04AAS1
Ilyobacter polytropus DSM 2926
Isosphaera pallida ATCC 43644
Kosmotoga olearia TBF 19.5.1
Ktedonobacter racemifer DSM 44963
Lactococcus lactis subsp. cremoris MG1363
Lawsonia intracellularis PHE/MN1-00
Lentisphaera araneosa
Leptolyngbya valderiana BDU 20041
Leptospira interrogans
Leptospirillum ferrooxidans
Leptotrichia hofstadii
Magnetococcus sp. MC-1
Maricaulis maris MCS10
Marinitoga piezophila KA3
Mariprofundus ferrooxydans PV-1
Meiothermus ruber DSM 1279
Meiothermus silvanus DSM 9946
Mesoplasma florum L1
Methylibium petroleiphilum PM1
Methylobacillus flagellatus KT
Microscilla marina

Mitsuokella multacida
Moorella thermoacetica
Mycoplasma agalactiae PG2
Mycoplasma pneumoniae M129
Myxococcus xanthus DK 1622
Nautilia profundicola AmH
Neisseria elongata ATCC 29315
Nitratifractor salsuginis
Nitratiruptor sp. SB155-2
Nitrosomonas eutropha C91
Nostoc punctiforme PCC 73102
Oceanithermus profundus
Opitutus terrae PB90-1
Parachlamydia acanthamoebae str. Hall's coccus
Pasteurella multocida subsp. multocida str. Pm70
Pedobacter sp. BAL39
Persephonella marina
Petrotoga mobilis SJ95
Photobacterium angustum S14
Pirellula staleyi DSM 6068
Planctomyces limnophilus DSM 3776
Porphyromonas gingivalis
Prevotella amnii CRIS 21A-A
Prochlorococcus marinus str. AS9601
Prosthecochloris aestuarii DSM 271
Pyramidobacter piscolens
Ralstonia eutropha H16
Rhizobium leguminosarum
Rhodobacter sphaeroides
Rhodopirellula baltica SH 1
Rhodospirillum rubrum ATCC 11170
Roseiflexus castenholzii DSM 13941
Rubrobacter xylanophilus DSM 9941
Salinibacter ruber DSM 13855
Sebaldella termitidis ATCC 33386
Solibacter usitatus Ellin6076
Sphingomonas wittichii RW1
Sphingopyxis alaskensis RB2256
Spirochaeta smaragdinae DSM 11293
Spiroplasma citri
Stenotrophomonas maltophilia K279a
Streptobacillus moniliformis DSM 12112
Streptococcus agalactiae
Sulfurihydrogenibium yellowstonense
Sulfurimonas denitrificans DSM 1251
Sulfurovum sp. NBC37-1
Syntrophobacter fumaroxidans MPOB
Syntrophus aciditrophicus SB
Terriglobus saanensis SP1PR4
Thermanaerovibrio acidaminovorans
Thermocrinis albus DSM 14484
Thermodesulfovibrio yellowstonii DSM 11347
Thermomicrobium roseum
Thermosipho africanus TCF52B
Thermotoga maritima MSB8
Thermovibrio ammonificans
Thermus thermophilus
Thiobacillus denitrificans ATCC 25259
Treponema brennaborense DSM 12168
Treponema denticola ATCC 35405
Truepera radiovictrix
Ureaplasma parvum serovar 1 str. ATCC 27813

	Verrucomicrobium spinosum DSM 4136<br>*Victivallis vadensis*<br>*Waddlia chondrophila WSU 86-1044*<br>*Xanthobacter autotrophicus Py2*<br>*Xanthomonas oryzae*
**Archaea**	*Acidilobus saccharovorans 345-15*<br>*Aciduliprofundum boonei T469*<br>*Aeropyrum pernix K1*<br>*Archaeoglobus fulgidus DSM 4304*<br>*Archaeoglobus profundus DSM 5631*<br>*Caldivirga maquilingensis IC-167*<br>*Candidatus Caldiarchaeum subterraneaum*<br>*Candidatus Korarchaeum cryptofilum OPF8*<br>*Candidatus Methanoregula boonei 6A8*<br>*Cenarchaeum symbiosum A*<br>*Desulfurococcus kamchatkensis 1221n*<br>*Ferroglobus placidus DSM 10642*<br>*Ferroplasma acidarmanus fer1*<br>*Halalkalicoccus jeotgali B3*<br>*Haloarcula marismortui ATCC 43049*<br>*Halobacterium salinarum*<br>*Haloferax volcanii DS2*<br>*Halogeometricum borinquense DSM 11551*<br>*Halomicrobium mukohataei DSM 12286*<br>*Haloquadratum walsbyi DSM 16790*<br>*Halorhabdus utahensis DSM 12940*<br>*Halorubrum lacusprofundi ATCC 49239*<br>*Haloterrigena turkmenica DSM 5511*<br>*Hyperthermus butylicus DSM 5456*<br>*Ignicoccus hospitalis KIN4/I*<br>*Ignisphaera aggregans DSM 17230*<br>*Metallosphaera sedula DSM 5348*<br>*Methanobrevibacter ruminantium M1*<br>*Methanobrevibacter smithii*<br>*Methanocaldococcus fervens AG86*<br>*Methanocaldococcus infernus ME*<br>*Methanocaldococcus jannaschii DSM 2661*<br>*Methanocaldococcus sp. FS406-22*<br>*Methanocaldococcus vulcanius M7*<br>*Methanocella paludicola SANAE*<br>*Methanococcoides burtonii DSM 6242*<br>*Methanococcoides burtonii DSM 6242*<br>*Methanococcus aeolicus Nankai-3*<br>*Methanococcus maripaludis*<br>*Methanococcus vannielii SB*<br>*Methanococcus voltae A3*<br>*Methanocorpusculum labreanum Z*<br>*Methanoculleus marisnigri JR1*<br>*Methanohalobium evestigatum Z-7303*<br>*Methanohalophilus mahii DSM 5219*<br>*Methanoplanus petrolearius DSM 11571*<br>*Methanopyrus kandleri AV19*<br>*Methanosaeta thermophila PT*<br>*Methanosarcina acetivorans C2A*<br>*Methanosarcina barkeri str. Fusaro*<br>*Methanosarcina mazei Go1*<br>*Methanosphaera stadtmanae DSM 3091*<br>*Methanosphaerula palustris E1-9c*<br>*Methanospirillum hungatei JF-1*<br>*Methanothermobacter marburgensis str. Marburg*<br>*Methanothermobacter thermautotrophicus Delta H*<br>*Methanothermococcus okinawensis IH1*

	Methanothermus fervidus
	Nanoarchaeum equitans Kin4-M
	Natrialba magadii ATCC 43099
	Natronomonas pharaonis DSM 2160
	Nitrosopumilus maritimus SCM1
	Picrophilus torridus DSM 9790
	Pyrobaculum aerophilum str. IM2
	Pyrobaculum arsenaticum DSM 13514
	Pyrobaculum calidifontis JCM 11548
	Pyrobaculum islandicum DSM 4184
	Pyrococcus abyssi GE5
	Pyrococcus furiosus DSM 3638
	Pyrococcus horikoshii OT3
	Staphylothermus hellenicus DSM 12710
	Staphylothermus marinus F1
	Sulfolobus acidocaldarius DSM 639
	Sulfolobus solfataricus P2
	Sulfolobus tokodaii str. 7
	Thermococcus barophilus MP
	Thermococcus gammatolerans EJ3
	Thermococcus kodakarensis KOD1
	Thermococcus onnurineus NA1
	Thermococcus sibiricus MM 739
	Thermococcus sp. AM4
	Thermofilum pendens Hrk 5
	Thermoplasma acidophilum DSM 1728
	Thermoplasma volcanium GSS1
	Thermoproteus neutrophilus V24Sta
	Thermoproteus tenax Kra1
	Thermosphaera aggregans DSM 11486
	Vulcanisaeta distributa DSM 14429
**Eukaryotes**	*Acanthamoeba castellanii*
	Arabidopsis thaliana
	Aureococcus anophagefferens
	Babesia bovis T2Bo
	Bigelowiella natans
	Blastocladiella emersonii
	Bryopsis hypnoides
	Chlamydomonas reinhardtii
	Chlorella vulgaris
	Chlorokybus atmophyticus
	Chromera velia
	Chrysodidymus synuroideus
	Cryptococcus neoformans var. grubii
	Cryptomonas paramecium
	Cryptosporidium hominis TU502
	Cyanidioschyzon merolae
	Cyanophora paradoxa
	Desmarestia viridis
	Dictyostelium discoideum AX4
	Durinskia baltica
	Eimeria tenella str. Houghton
	Emiliania huxleyi
	Encephalitozoon cuniculi GB-M1
	Entamoeba histolytica HM-1:IMSS
	Euglena gracilis
	Giardia intestinalis
	Glaucocystis nostochinearum
	Glomus irregulare
	Gracilaria chilensis
	Guillardia theta
	Haemoproteus columbae

Hartmannella vermiformis
Hemiselmis andersenii
Igernella notabilis
Kryptoperidinium foliaceum
Leishmania major
Leucocytozoon majoris
Malawimonas jakobiformis
Mesostigma viride
Micromonas pusilla CCMP1545
Monosiga brevicollis ATCC 50154
Naegleria gruberi
Nephroselmis olivacea
Ochromonas danica
Odontella sinensis
Pan troglodytes verus
Parahaemoproteus vireonis
Paramecium tetraurelia strain d4-2
Paulinella chromatophora
Perkinsus marinus
Phakopsora pachyrhizi
Physarum polycephalum
Phytophthora infestans T30-4
Plasmodium falciparum 3D7
Porphyra purpurea
Reclinomonas americana
Rhodomonas salina
Saccharomyces cerevisiae A364A
Schizosaccharomyces pombe 972h
Staurastrum punctulatum
Synedra acus Cafeteria roenbergensis
Talaromyces stipitatus ATCC 10500
Tetrahymena thermophila
Thalassiosira oceanica CCMP1005
Theileria annulata strain Ankara
Toxoplasma gondii ME49
Trichomonas vaginalis G3
Trichoplax adhaerens
Trypanosoma cruzi
Ustilago maydis 521

SUPPLEMENTARY FIGURE 1: Complete short-chain IPPS phylogeny reconstructed using 136 representative sequences and 244 conserved sites.

Suppl. Figure 1.

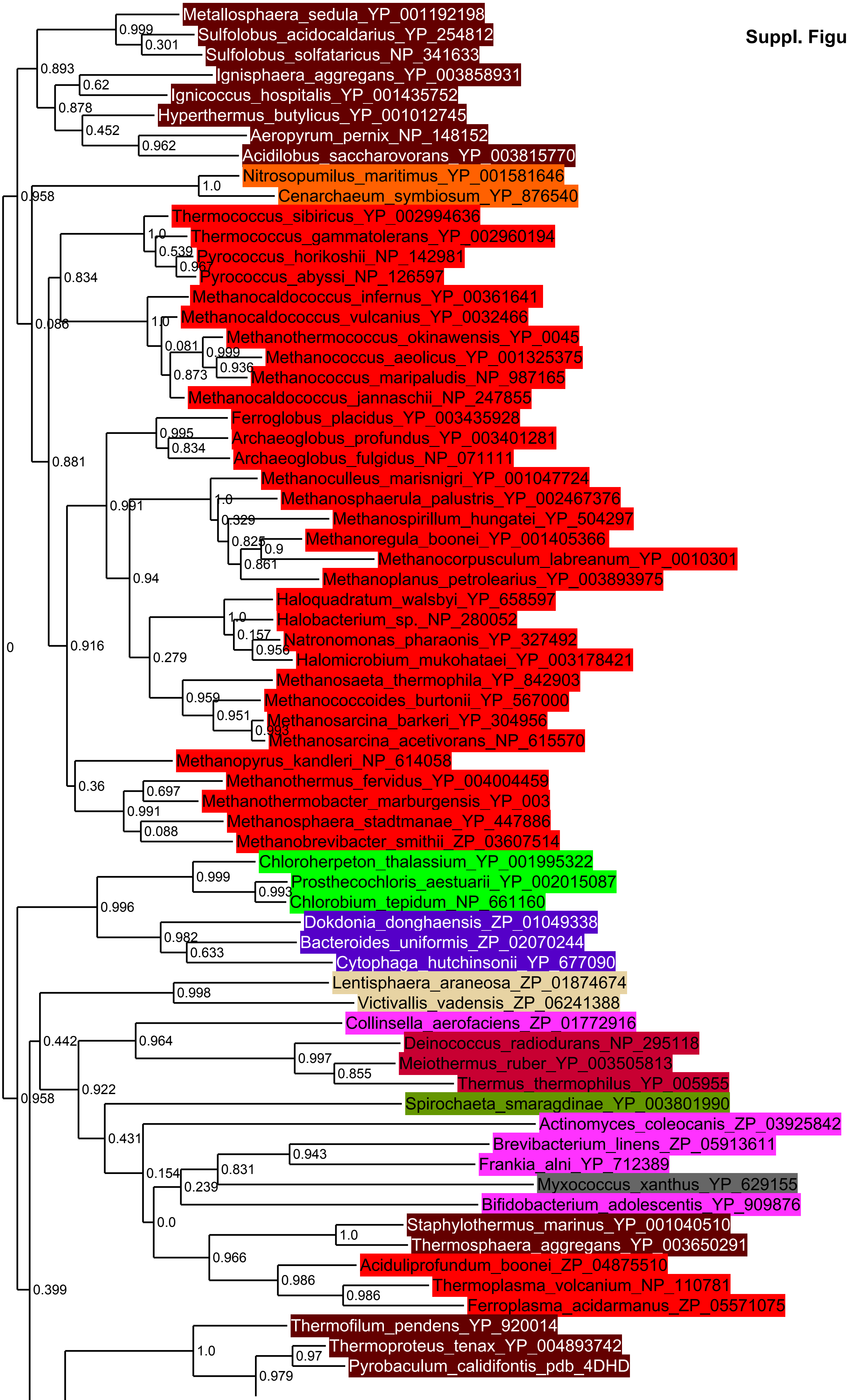

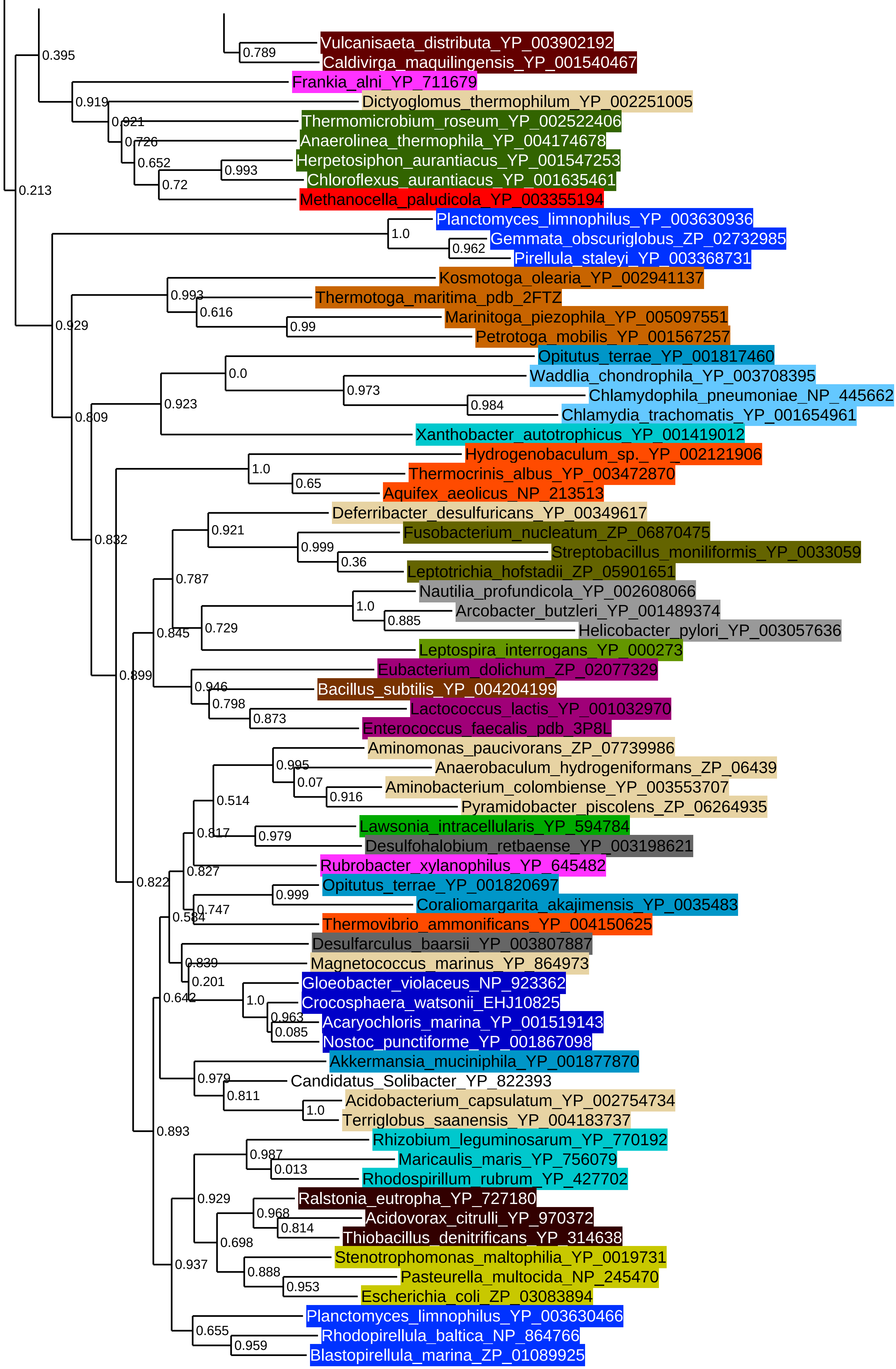

Vulcanisaeta_distributa_YP_003902192
Caldivirga_maquilingensis_YP_001540467
Frankia_alni_YP_711679
Dictyoglomus_thermophilum_YP_002251005
Thermomicrobium_roseum_YP_002522406
Anaerolinea_thermophila_YP_004174678
Herpetosiphon_aurantiacus_YP_001547253
Chloroflexus_aurantiacus_YP_001635461
Methanocella_paludicola_YP_003355194
Planctomyces_limnophilus_YP_003630936
Gemmata_obscuriglobus_ZP_02732985
Pirellula_staleyi_YP_003368731
Kosmotoga_olearia_YP_002941137
Thermotoga_maritima_pdb_2FTZ
Marinitoga_piezophila_YP_005097551
Petrotoga_mobilis_YP_001567257
Opitutus_terrae_YP_001817460
Waddlia_chondrophila_YP_003708395
Chlamydophila_pneumoniae_NP_445662
Chlamydia_trachomatis_YP_001654961
Xanthobacter_autotrophicus_YP_001419012
Hydrogenobaculum_sp._YP_002121906
Thermocrinis_albus_YP_003472870
Aquifex_aeolicus_NP_213513
Deferribacter_desulfuricans_YP_00349617
Fusobacterium_nucleatum_ZP_06870475
Streptobacillus_moniliformis_YP_0033059
Leptotrichia_hofstadii_ZP_05901651
Nautilia_profundicola_YP_002608066
Arcobacter_butzleri_YP_001489374
Helicobacter_pylori_YP_003057636
Leptospira_interrogans_YP_000273
Eubacterium_dolichum_ZP_02077329
Bacillus_subtilis_YP_004204199
Lactococcus_lactis_YP_001032970
Enterococcus_faecalis_pdb_3P8L
Aminomonas_paucivorans_ZP_07739986
Anaerobaculum_hydrogeniformans_ZP_06439
Aminobacterium_colombiense_YP_003553707
Pyramidobacter_piscolens_ZP_06264935
Lawsonia_intracellularis_YP_594784
Desulfohalobium_retbaense_YP_003198621
Rubrobacter_xylanophilus_YP_645482
Opitutus_terrae_YP_001820697
Coraliomargarita_akajimensis_YP_0035483
Thermovibrio_ammonificans_YP_004150625
Desulfarculus_baarsii_YP_003807887
Magnetococcus_marinus_YP_864973
Gloeobacter_violaceus_NP_923362
Crocosphaera_watsonii_EHJ10825
Acaryochloris_marina_YP_001519143
Nostoc_punctiforme_YP_001867098
Akkermansia_muciniphila_YP_001877870
Candidatus_Solibacter_YP_822393
Acidobacterium_capsulatum_YP_002754734
Terriglobus_saanensis_YP_004183737
Rhizobium_leguminosarum_YP_770192
Maricaulis_maris_YP_756079
Rhodospirillum_rubrum_YP_427702
Ralstonia_eutropha_YP_727180
Acidovorax_citrulli_YP_970372
Thiobacillus_denitrificans_YP_314638
Stenotrophomonas_maltophilia_YP_0019731
Pasteurella_multocida_NP_245470
Escherichia_coli_ZP_03083894
Planctomyces_limnophilus_YP_003630466
Rhodopirellula_baltica_NP_864766
Blastopirellula_marina_ZP_01089925
0.395
0.789
0.919
0.921
0.726
0.652
0.993
0.72
0.213
1.0
0.962
0.993
0.616
0.99
0.929
0.0
0.973
0.984
0.923
0.809
1.0
0.65
0.921
0.999
0.36
0.832
0.787
1.0
0.885
0.729
0.845
0.899
0.946
0.798
0.873
0.995
0.07
0.916
0.514
0.817
0.979
0.827
0.822
0.999
0.747
0.584
0.839
0.201
1.0
0.642
0.963
0.085
0.979
0.811
1.0
0.893
0.987
0.013
0.929
0.968
0.814
0.698
0.937
0.888
0.953
0.655
0.959

SUPPLEMENTARY FIGURE 2: Complete long-chain IPPS phylogeny reconstructed using 218 representative sequences and 241 conserved sites.

Suppl. Figure 2.

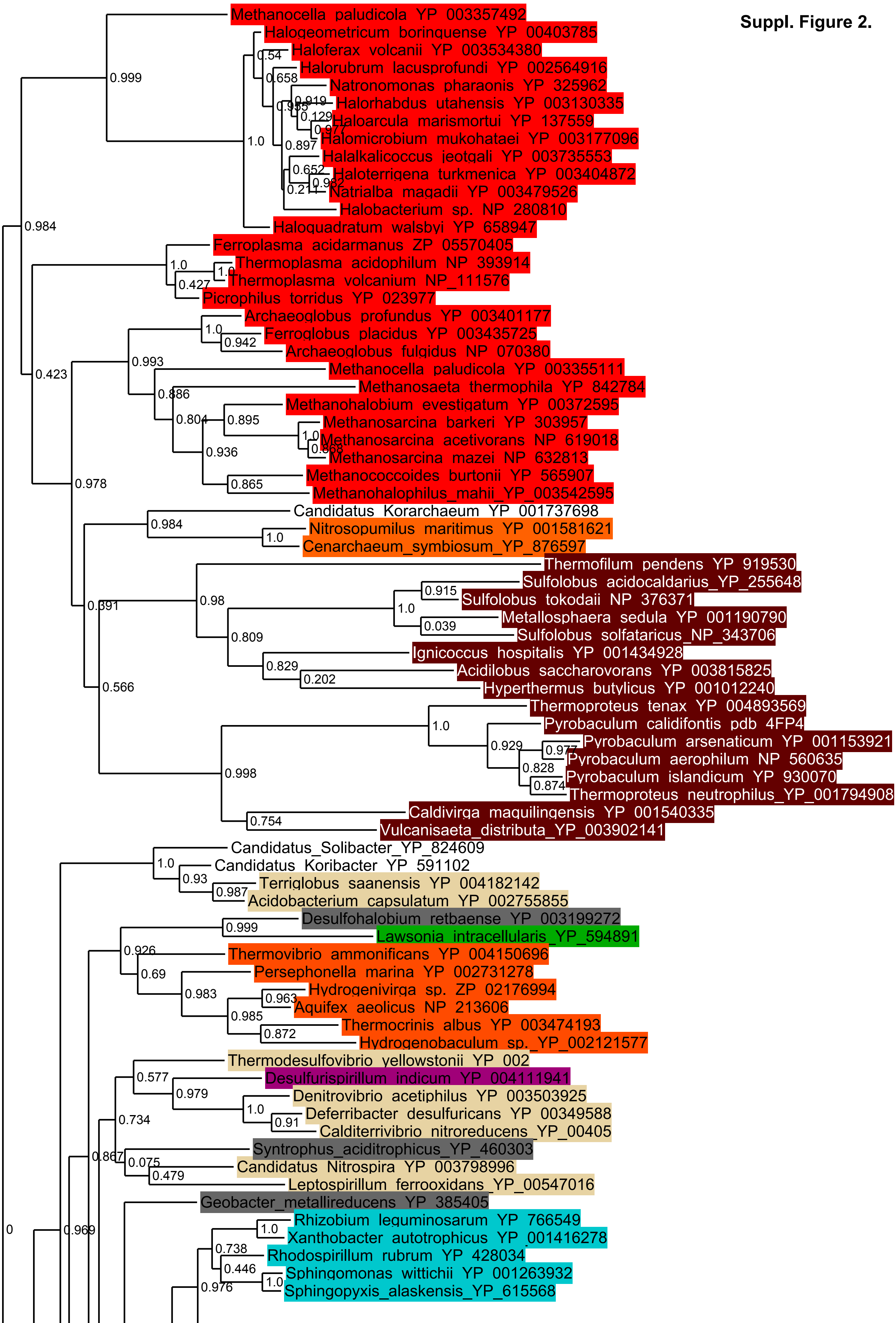

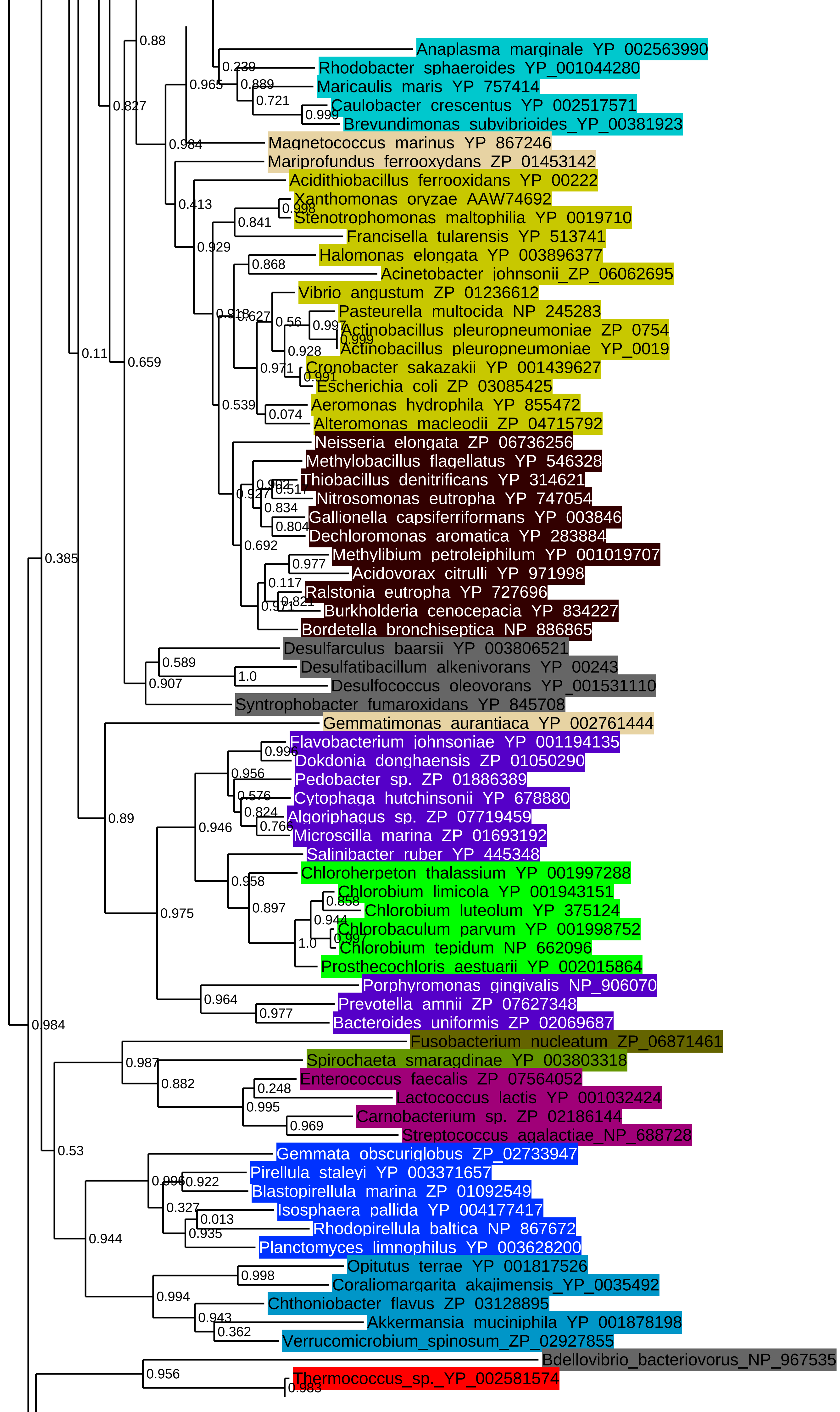

0.88
Anaplasma_marginale_YP_002563990
0.239
Rhodobacter_sphaeroides_YP_001044280
0.965
0.889
Maricaulis_maris_YP_757414
0.827
0.721
Caulobacter_crescentus_YP_002517571
0.999
Brevundimonas_subvibrioides_YP_00381923
0.984
Magnetococcus_marinus_YP_867246
Mariprofundus_ferrooxydans_ZP_01453142
Acidithiobacillus_ferrooxidans_YP_00222
0.413
Xanthomonas_oryzae_AAW74692
0.998
0.841
Stenotrophomonas_maltophilia_YP_0019710
Francisella_tularensis_YP_513741
0.929
Halomonas_elongata_YP_003896377
0.868
Acinetobacter_johnsonii_ZP_06062695
Vibrio_angustum_ZP_01236612
0.918
0.627
Pasteurella_multocida_NP_245283
0.56
0.997
Actinobacillus_pleuropneumoniae_ZP_0754
0.999
0.11
0.659
0.928
Actinobacillus_pleuropneumoniae_YP_0019
0.971
Cronobacter_sakazakii_YP_001439627
0.991
Escherichia_coli_ZP_03085425
0.539
Aeromonas_hydrophila_YP_855472
0.074
Alteromonas_macleodii_ZP_04715792
Neisseria_elongata_ZP_06736256
Methylobacillus_flagellatus_YP_546328
0.904
Thiobacillus_denitrificans_YP_314621
0.927
0.517
Nitrosomonas_eutropha_YP_747054
0.834
Gallionella_capsiferriformans_YP_003846
0.804
Dechloromonas_aromatica_YP_283884
0.692
Methylibium_petroleiphilum_YP_001019707
0.385
0.977
Acidovorax_citrulli_YP_971998
0.117
Ralstonia_eutropha_YP_727696
0.821
0.971
Burkholderia_cenocepacia_YP_834227
Bordetella_bronchiseptica_NP_886865
Desulfarculus_baarsii_YP_003806521
0.589
Desulfatibacillum_alkenivorans_YP_00243
1.0
0.907
Desulfococcus_oleovorans_YP_001531110
Syntrophobacter_fumaroxidans_YP_845708
Gemmatimonas_aurantiaca_YP_002761444
Flavobacterium_johnsoniae_YP_001194135
0.996
Dokdonia_donghaensis_ZP_01050290
0.956
Pedobacter_sp._ZP_01886389
0.576
Cytophaga_hutchinsonii_YP_678880
0.824
0.89
Algoriphagus_sp._ZP_07719459
0.946
0.766
Microscilla_marina_ZP_01693192
Salinibacter_ruber_YP_445348
Chloroherpeton_thalassium_YP_001997288
0.958
Chlorobium_limicola_YP_001943151
0.858
0.897
Chlorobium_luteolum_YP_375124
0.975
0.944
Chlorobaculum_parvum_YP_001998752
0.997
1.0
Chlorobium_tepidum_NP_662096
Prosthecochloris_aestuarii_YP_002015864
Porphyromonas_gingivalis_NP_906070
0.964
Prevotella_amnii_ZP_07627348
0.977
Bacteroides_uniformis_ZP_02069687
0.984
Fusobacterium_nucleatum_ZP_06871461
0.987
Spirochaeta_smaragdinae_YP_003803318
0.882
Enterococcus_faecalis_ZP_07564052
0.248
Lactococcus_lactis_YP_001032424
0.995
Carnobacterium_sp._ZP_02186144
0.969
Streptococcus_agalactiae_NP_688728
0.53
Gemmata_obscuriglobus_ZP_02733947
Pirellula_staleyi_YP_003371657
0.996
0.922
Blastopirellula_marina_ZP_01092549
0.327
Isosphaera_pallida_YP_004177417
0.013
0.935
Rhodopirellula_baltica_NP_867672
0.944
Planctomyces_limnophilus_YP_003628200
Opitutus_terrae_YP_001817526
0.998
Coraliomargarita_akajimensis_YP_0035492
0.994
Chthoniobacter_flavus_ZP_03128895
0.943
Akkermansia_muciniphila_YP_001878198
0.362
Verrucomicrobium_spinosum_ZP_02927855
Bdellovibrio_bacteriovorus_NP_967535
0.956
Thermococcus_sp._YP_002581574
0.983

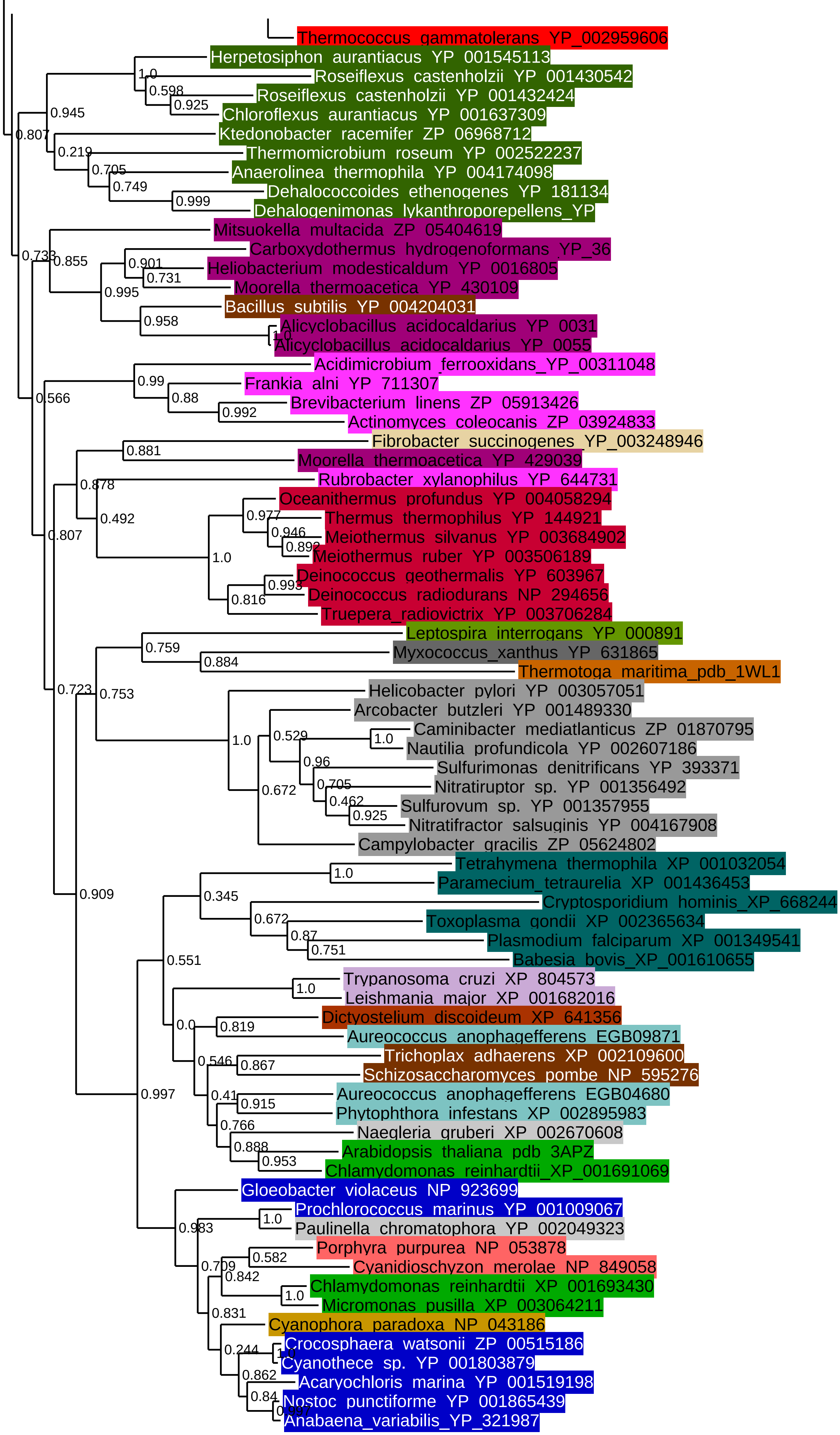
Thermococcus_gammatolerans_YP_002959606
Herpetosiphon_aurantiacus_YP_001545113
Roseiflexus_castenholzii_YP_001430542
Roseiflexus_castenholzii_YP_001432424
Chloroflexus_aurantiacus_YP_001637309
Ktedonobacter_racemifer_ZP_06968712
Thermomicrobium_roseum_YP_002522237
Anaerolinea_thermophila_YP_004174098
Dehalococcoides_ethenogenes_YP_181134
Dehalogenimonas_lykanthroporepellens_YP
Mitsuokella_multacida_ZP_05404619
Carboxydothermus_hydrogenoformans_YP_36
Heliobacterium_modesticaldum_YP_0016805
Moorella_thermoacetica_YP_430109
Bacillus_subtilis_YP_004204031
Alicyclobacillus_acidocaldarius_YP_0031
Alicyclobacillus_acidocaldarius_YP_0055
Acidimicrobium_ferrooxidans_YP_00311048
Frankia_alni_YP_711307
Brevibacterium_linens_ZP_05913426
Actinomyces_coleocanis_ZP_03924833
Fibrobacter_succinogenes_YP_003248946
Moorella_thermoacetica_YP_429039
Rubrobacter_xylanophilus_YP_644731
Oceanithermus_profundus_YP_004058294
Thermus_thermophilus_YP_144921
Meiothermus_silvanus_YP_003684902
Meiothermus_ruber_YP_003506189
Deinococcus_geothermalis_YP_603967
Deinococcus_radiodurans_NP_294656
Truepera_radiovictrix_YP_003706284
Leptospira_interrogans_YP_000891
Myxococcus_xanthus_YP_631865
Thermotoga_maritima_pdb_1WL1
Helicobacter_pylori_YP_003057051
Arcobacter_butzleri_YP_001489330
Caminibacter_mediatlanticus_ZP_01870795
Nautilia_profundicola_YP_002607186
Sulfurimonas_denitrificans_YP_393371
Nitratiruptor_sp._YP_001356492
Sulfurovum_sp._YP_001357955
Nitratifractor_salsuginis_YP_004167908
Campylobacter_gracilis_ZP_05624802
Tetrahymena_thermophila_XP_001032054
Paramecium_tetraurelia_XP_001436453
Cryptosporidium_hominis_XP_668244
Toxoplasma_gondii_XP_002365634
Plasmodium_falciparum_XP_001349541
Babesia_bovis_XP_001610655
Trypanosoma_cruzi_XP_804573
Leishmania_major_XP_001682016
Dictyostelium_discoideum_XP_641356
Aureococcus_anophagefferens_EGB09871
Trichoplax_adhaerens_XP_002109600
Schizosaccharomyces_pombe_NP_595276
Aureococcus_anophagefferens_EGB04680
Phytophthora_infestans_XP_002895983
Naegleria_gruberi_XP_002670608
Arabidopsis_thaliana_pdb_3APZ
Chlamydomonas_reinhardtii_XP_001691069
Gloeobacter_violaceus_NP_923699
Prochlorococcus_marinus_YP_001009067
Paulinella_chromatophora_YP_002049323
Porphyra_purpurea_NP_053878
Cyanidioschyzon_merolae_NP_849058
Chlamydomonas_reinhardtii_XP_001693430
Micromonas_pusilla_XP_003064211
Cyanophora_paradoxa_NP_043186
Crocosphaera_watsonii_ZP_00515186
Cyanothece_sp._YP_001803879
Acaryochloris_marina_YP_001519198
Nostoc_punctiforme_YP_001865439
Anabaena_variabilis_YP_321987
1.0
0.598
0.925
0.945
0.807
0.219
0.705
0.749
0.999
0.733
0.855
0.901
0.731
0.995
0.958
1.0
0.99
0.566
0.88
0.992
0.881
0.878
0.492
0.807
0.977
0.946
0.892
1.0
0.993
0.816
0.759
0.884
0.723
0.753
1.0
0.529
1.0
0.96
0.705
0.672
0.462
0.925
1.0
0.345
0.909
0.672
0.87
0.751
0.551
1.0
0.0
0.819
0.546
0.867
0.997
0.41
0.915
0.766
0.888
0.953
1.0
0.983
0.582
0.709
0.842
1.0
0.831
0.244
1.0
0.862
0.84
0.997

SUPPLEMENTARY FIGURE 3: GGGPS phylogeny reconstructed using 135 representative sequences and 176 conserved sites.

Suppl. Figure 3.

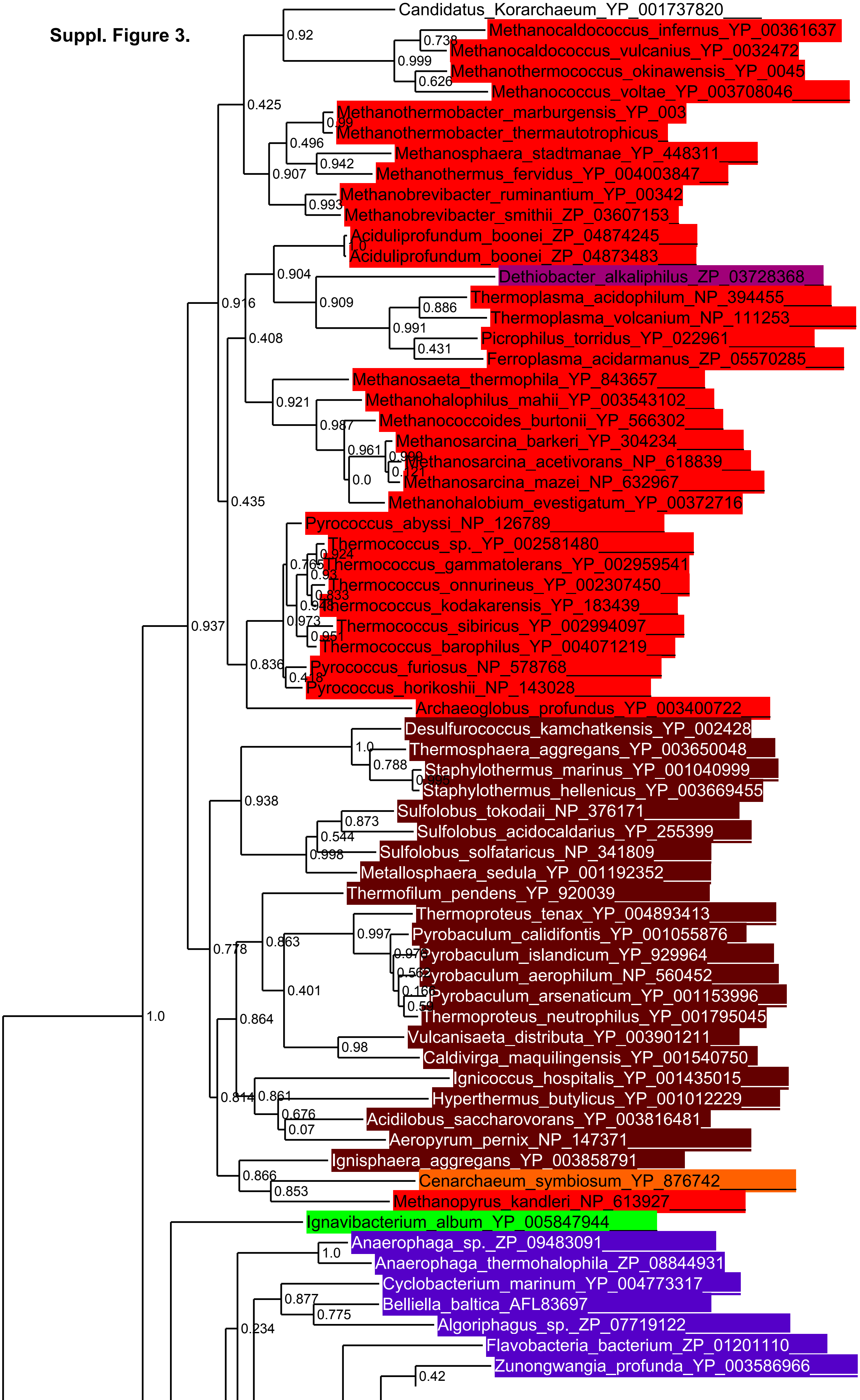

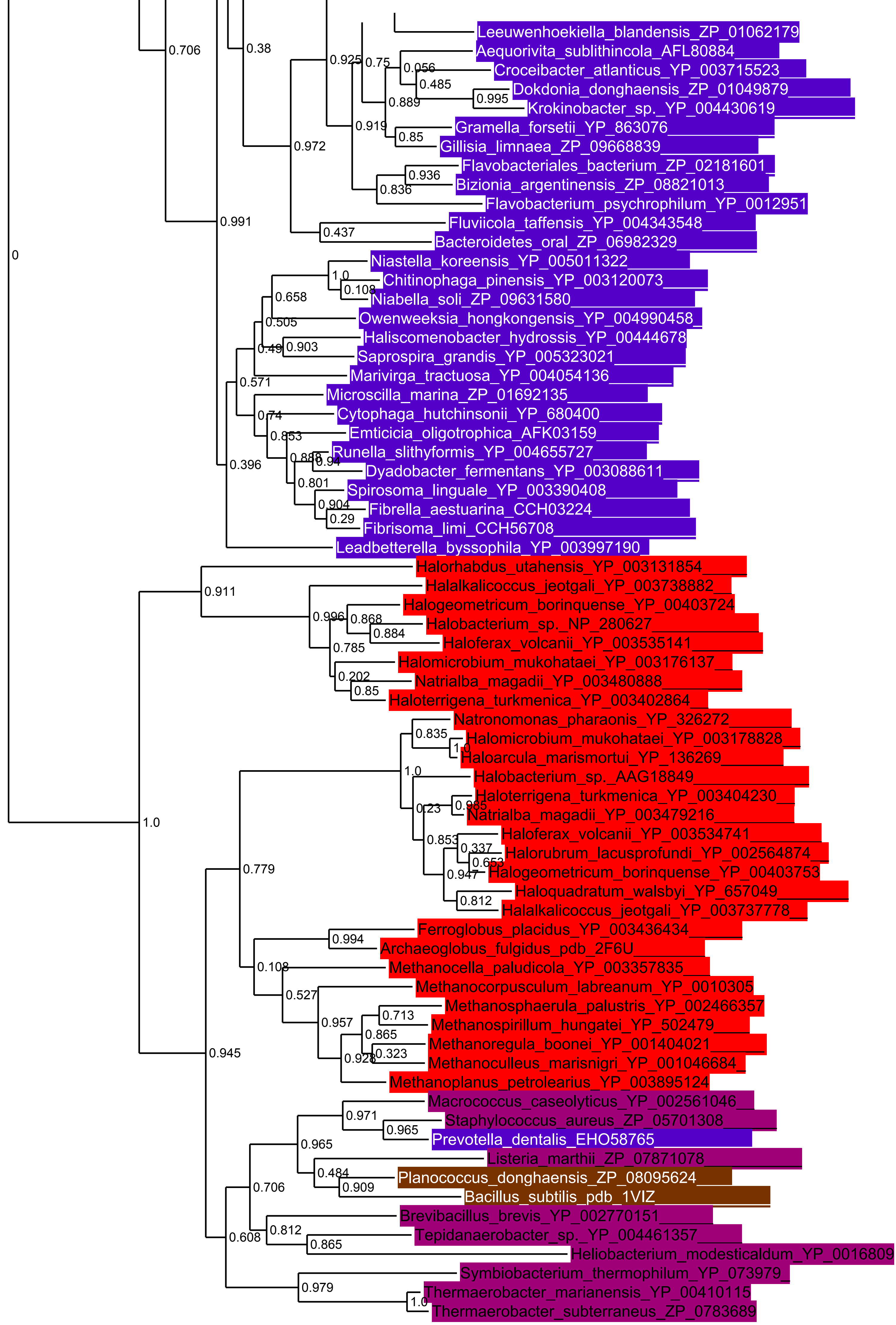
Leeuwenhoekiella_blandensis_ZP_01062179
Aequorivita_sublithincola_AFL80884
Croceibacter_atlanticus_YP_003715523
Dokdonia_donghaensis_ZP_01049879
Krokinobacter_sp._YP_004430619
Gramella_forsetii_YP_863076
Gillisia_limnaea_ZP_09668839
Flavobacteriales_bacterium_ZP_02181601
Bizionia_argentinensis_ZP_08821013
Flavobacterium_psychrophilum_YP_0012951
Fluviicola_taffensis_YP_004343548
Bacteroidetes_oral_ZP_06982329
Niastella_koreensis_YP_005011322
Chitinophaga_pinensis_YP_003120073
Niabella_soli_ZP_09631580
Owenweeksia_hongkongensis_YP_004990458
Haliscomenobacter_hydrossis_YP_00444678
Saprospira_grandis_YP_005323021
Marivirga_tractuosa_YP_004054136
Microscilla_marina_ZP_01692135
Cytophaga_hutchinsonii_YP_680400
Emticicia_oligotrophica_AFK03159
Runella_slithyformis_YP_004655727
Dyadobacter_fermentans_YP_003088611
Spirosoma_linguale_YP_003390408
Fibrella_aestuarina_CCH03224
Fibrisoma_limi_CCH56708
Leadbetterella_byssophila_YP_003997190
Halorhabdus_utahensis_YP_003131854
Halalkalicoccus_jeotgali_YP_003738882
Halogeometricum_borinquense_YP_00403724
Halobacterium_sp._NP_280627
Haloferax_volcanii_YP_003535141
Halomicrobium_mukohataei_YP_003176137
Natrialba_magadii_YP_003480888
Haloterrigena_turkmenica_YP_003402864
Natronomonas_pharaonis_YP_326272
Halomicrobium_mukohataei_YP_003178828
Haloarcula_marismortui_YP_136269
Halobacterium_sp._AAG18849
Haloterrigena_turkmenica_YP_003404230
Natrialba_magadii_YP_003479216
Haloferax_volcanii_YP_003534741
Halorubrum_lacusprofundi_YP_002564874
Halogeometricum_borinquense_YP_00403753
Haloquadratum_walsbyi_YP_657049
Halalkalicoccus_jeotgali_YP_003737778
Ferroglobus_placidus_YP_003436434
Archaeoglobus_fulgidus_pdb_2F6U
Methanocella_paludicola_YP_003357835
Methanocorpusculum_labreanum_YP_0010305
Methanosphaerula_palustris_YP_002466357
Methanospirillum_hungatei_YP_502479
Methanoregula_boonei_YP_001404021
Methanoculleus_marisnigri_YP_001046684
Methanoplanus_petrolearius_YP_003895124
Macrococcus_caseolyticus_YP_002561046
Staphylococcus_aureus_ZP_05701308
Prevotella_dentalis_EHO58765
Listeria_marthii_ZP_07871078
Planococcus_donghaensis_ZP_08095624
Bacillus_subtilis_pdb_1VIZ
Brevibacillus_brevis_YP_002770151
Tepidanaerobacter_sp._YP_004461357
Heliobacterium_modesticaldum_YP_0016809
Symbiobacterium_thermophilum_YP_073979
Thermaerobacter_marianensis_YP_00410115
Thermaerobacter_subterraneus_ZP_0783689
0.706
0.38
0.925
0.75
0.056
0.485
0.995
0.889
0.919
0.85
0.972
0.936
0.836
0.991
0.437
0
1.0
0.108
0.658
0.505
0.903
0.49
0.571
0.74
0.853
0.888
0.94
0.396
0.801
0.904
0.29
0.911
0.996
0.868
0.884
0.785
0.202
0.85
0.835
1.0
1.0
0.23
0.985
0.853
0.337
0.653
0.947
0.812
1.0
0.779
0.994
0.108
0.527
0.713
0.957
0.865
0.323
0.928
0.945
0.971
0.965
0.965
0.484
0.909
0.706
0.812
0.865
0.608
0.979
1.0

SUPPLEMENTARY FIGURE 4: DGGGPS phylogeny reconstructed using 278 representative sequences and 114 conserved sites.

Suppl. Figure 4.

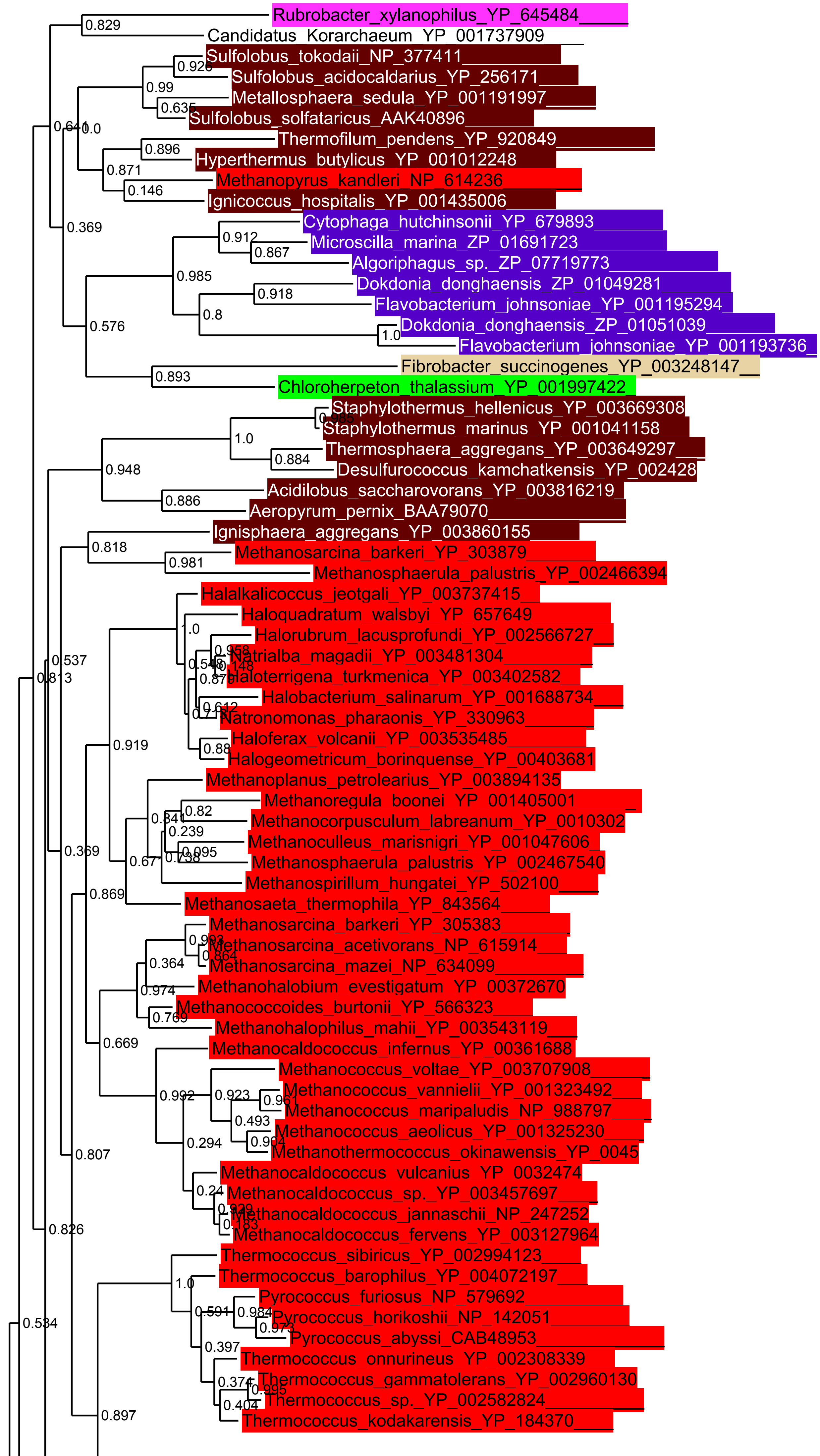

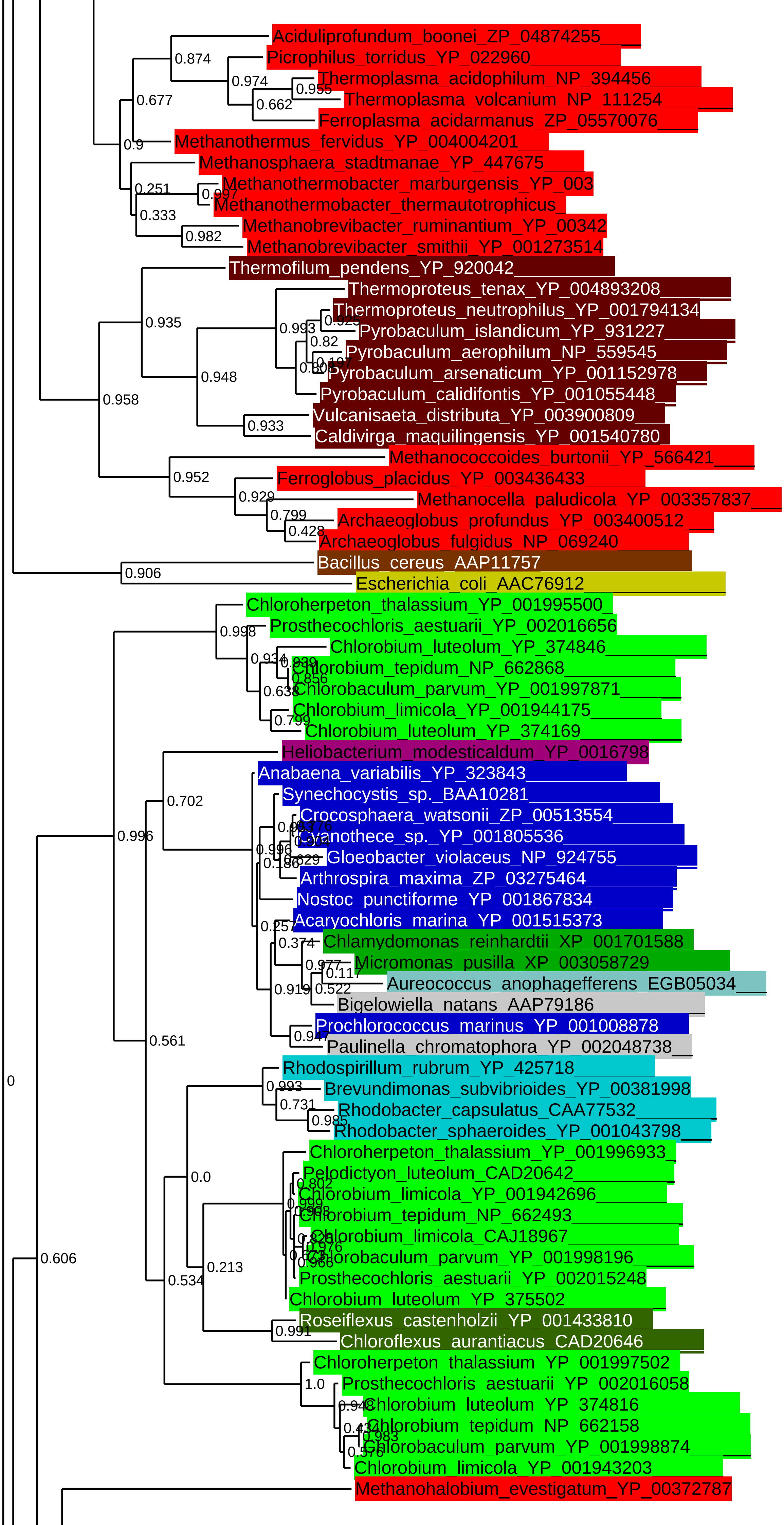

Aciduliprofundum_boonei_ZP_04874255
Picrophilus_torridus_YP_022960
Thermoplasma_acidophilum_NP_394456
Thermoplasma_volcanium_NP_111254
Ferroplasma_acidarmanus_ZP_05570076
Methanothermus_fervidus_YP_004004201
Methanosphaera_stadtmanae_YP_447675
Methanothermobacter_marburgensis_YP_003
Methanothermobacter_thermautotrophicus
Methanobrevibacter_ruminantium_YP_00342
Methanobrevibacter_smithii_YP_001273514
Thermofilum_pendens_YP_920042
Thermoproteus_tenax_YP_004893208
Thermoproteus_neutrophilus_YP_001794134
Pyrobaculum_islandicum_YP_931227
Pyrobaculum_aerophilum_NP_559545
Pyrobaculum_arsenaticum_YP_001152978
Pyrobaculum_calidifontis_YP_001055448
Vulcanisaeta_distributa_YP_003900809
Caldivirga_maquilingensis_YP_001540780
Methanococcoides_burtonii_YP_566421
Ferroglobus_placidus_YP_003436433
Methanocella_paludicola_YP_003357837
Archaeoglobus_profundus_YP_003400512
Archaeoglobus_fulgidus_NP_069240
Bacillus_cereus_AAP11757
Escherichia_coli_AAC76912
Chloroherpeton_thalassium_YP_001995500
Prosthecochloris_aestuarii_YP_002016656
Chlorobium_luteolum_YP_374846
Chlorobium_tepidum_NP_662868
Chlorobaculum_parvum_YP_001997871
Chlorobium_limicola_YP_001944175
Chlorobium_luteolum_YP_374169
Heliobacterium_modesticaldum_YP_0016798
Anabaena_variabilis_YP_323843
Synechocystis_sp._BAA10281
Crocosphaera_watsonii_ZP_00513554
Cyanothece_sp._YP_001805536
Gloeobacter_violaceus_NP_924755
Arthrospira_maxima_ZP_03275464
Nostoc_punctiforme_YP_001867834
Acaryochloris_marina_YP_001515373
Chlamydomonas_reinhardtii_XP_001701588
Micromonas_pusilla_XP_003058729
Aureococcus_anophagefferens_EGB05034
Bigelowiella_natans_AAP79186
Prochlorococcus_marinus_YP_001008878
Paulinella_chromatophora_YP_002048738
Rhodospirillum_rubrum_YP_425718
Brevundimonas_subvibrioides_YP_00381998
Rhodobacter_capsulatus_CAA77532
Rhodobacter_sphaeroides_YP_001043798
Chloroherpeton_thalassium_YP_001996933
Pelodictyon_luteolum_CAD20642
Chlorobium_limicola_YP_001942696
Chlorobium_tepidum_NP_662493
Chlorobium_limicola_CAJ18967
Chlorobaculum_parvum_YP_001998196
Prosthecochloris_aestuarii_YP_002015248
Chlorobium_luteolum_YP_375502
Roseiflexus_castenholzii_YP_001433810
Chloroflexus_aurantiacus_CAD20646
Chloroherpeton_thalassium_YP_001997502
Prosthecochloris_aestuarii_YP_002016058
Chlorobium_luteolum_YP_374816
Chlorobium_tepidum_NP_662158
Chlorobaculum_parvum_YP_001998874
Chlorobium_limicola_YP_001943203
Methanohalobium_evestigatum_YP_00372787

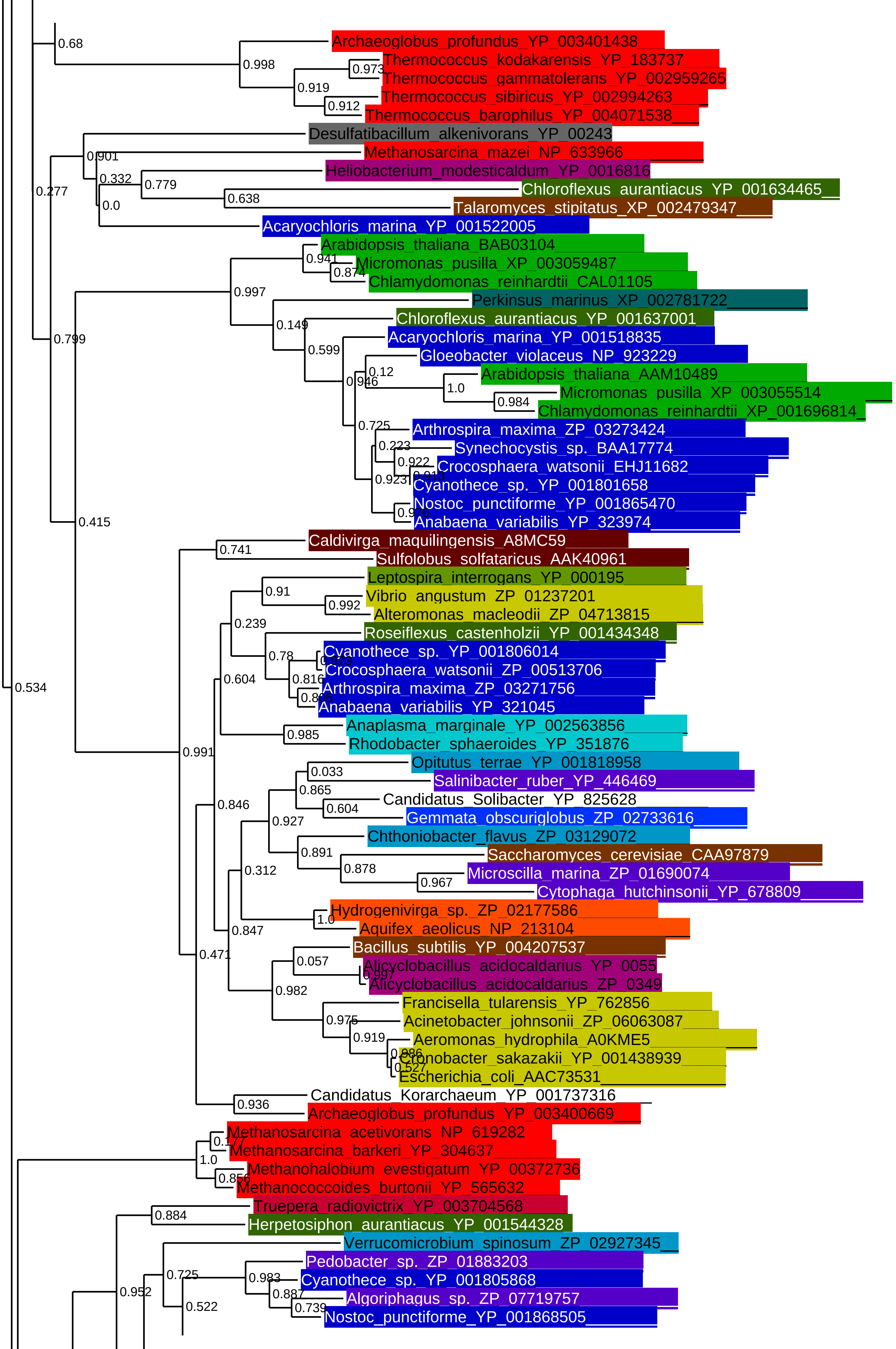

Archaeoglobus_profundus_YP_003401438
Thermococcus_kodakarensis_YP_183737
Thermococcus_gammatolerans_YP_002959265
Thermococcus_sibiricus_YP_002994263
Thermococcus_barophilus_YP_004071538
Desulfatibacillum_alkenivorans_YP_00243
Methanosarcina_mazei_NP_633966
Heliobacterium_modesticaldum_YP_0016816
Chloroflexus_aurantiacus_YP_001634465
Talaromyces_stipitatus_XP_002479347
Acaryochloris_marina_YP_001522005
Arabidopsis_thaliana_BAB03104
Micromonas_pusilla_XP_003059487
Chlamydomonas_reinhardtii_CAL01105
Perkinsus_marinus_XP_002781722
Chloroflexus_aurantiacus_YP_001637001
Acaryochloris_marina_YP_001518835
Gloeobacter_violaceus_NP_923229
Arabidopsis_thaliana_AAM10489
Micromonas_pusilla_XP_003055514
Chlamydomonas_reinhardtii_XP_001696814
Arthrospira_maxima_ZP_03273424
Synechocystis_sp._BAA17774
Crocosphaera_watsonii_EHJ11682
Cyanothece_sp._YP_001801658
Nostoc_punctiforme_YP_001865470
Anabaena_variabilis_YP_323974
Caldivirga_maquilingensis_A8MC59
Sulfolobus_solfataricus_AAK40961
Leptospira_interrogans_YP_000195
Vibrio_angustum_ZP_01237201
Alteromonas_macleodii_ZP_04713815
Roseiflexus_castenholzii_YP_001434348
Cyanothece_sp._YP_001806014
Crocosphaera_watsonii_ZP_00513706
Arthrospira_maxima_ZP_03271756
Anabaena_variabilis_YP_321045
Anaplasma_marginale_YP_002563856
Rhodobacter_sphaeroides_YP_351876
Opitutus_terrae_YP_001818958
Salinibacter_ruber_YP_446469
Candidatus_Solibacter_YP_825628
Gemmata_obscuriglobus_ZP_02733616
Chthoniobacter_flavus_ZP_03129072
Saccharomyces_cerevisiae_CAA97879
Microscilla_marina_ZP_01690074
Cytophaga_hutchinsonii_YP_678809
Hydrogenivirga_sp._ZP_02177586
Aquifex_aeolicus_NP_213104
Bacillus_subtilis_YP_004207537
Alicyclobacillus_acidocaldarius_YP_0055
Alicyclobacillus_acidocaldarius_ZP_0349
Francisella_tularensis_YP_762856
Acinetobacter_johnsonii_ZP_06063087
Aeromonas_hydrophila_A0KME5
Cronobacter_sakazakii_YP_001438939
Escherichia_coli_AAC73531
Candidatus_Korarchaeum_YP_001737316
Archaeoglobus_profundus_YP_003400669
Methanosarcina_acetivorans_NP_619282
Methanosarcina_barkeri_YP_304637
Methanohalobium_evestigatum_YP_00372736
Methanococcoides_burtonii_YP_565632
Truepera_radiovictrix_YP_003704568
Herpetosiphon_aurantiacus_YP_001544328
Verrucomicrobium_spinosum_ZP_02927345
Pedobacter_sp._ZP_01883203
Cyanothece_sp._YP_001805868
Algoriphagus_sp._ZP_07719757
Nostoc_punctiforme_YP_001868505

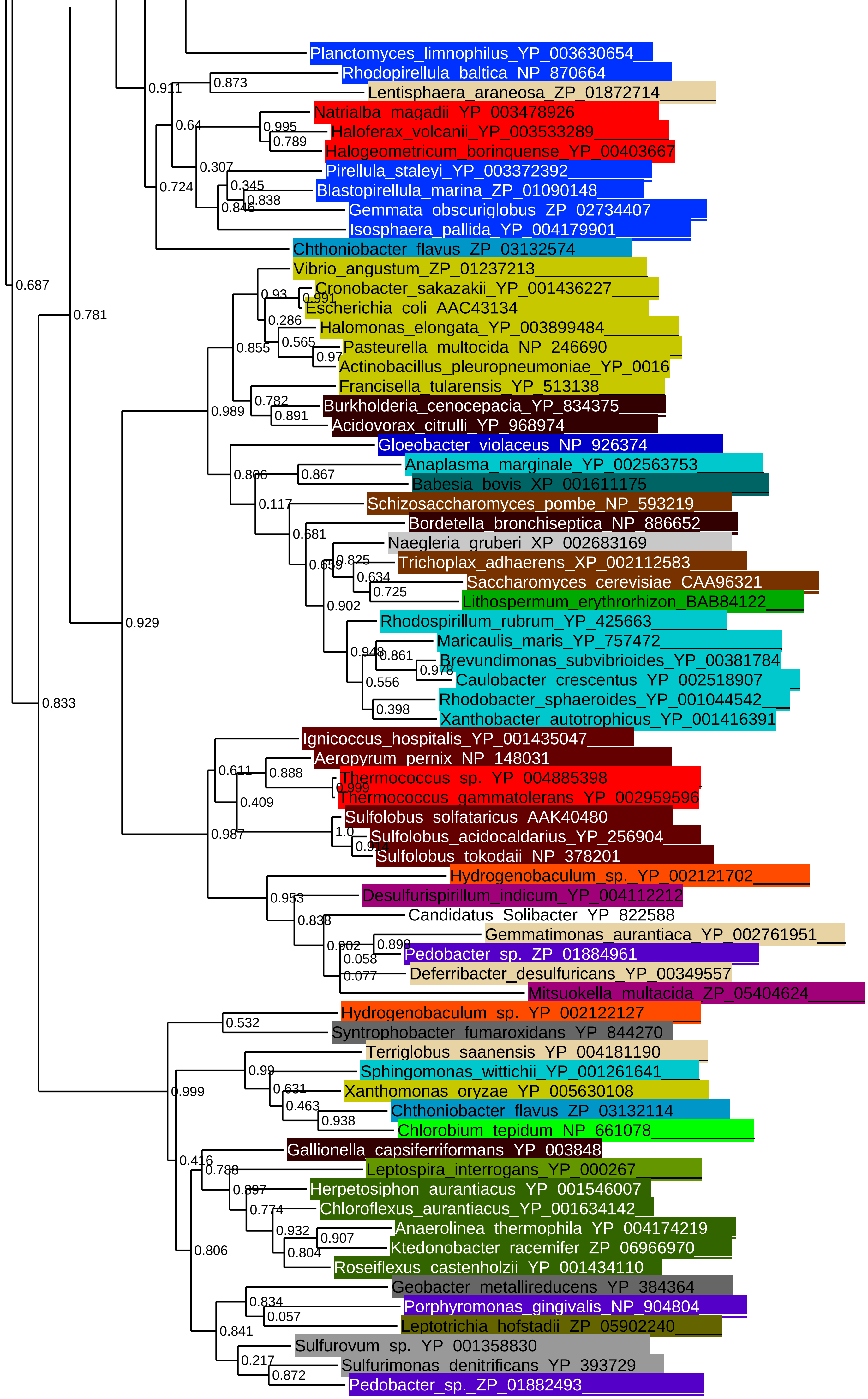
Planctomyces_limnophilus_YP_003630654
Rhodopirellula_baltica_NP_870664
Lentisphaera_araneosa_ZP_01872714
Natrialba_magadii_YP_003478926
Haloferax_volcanii_YP_003533289
Halogeometricum_borinquense_YP_00403667
Pirellula_staleyi_YP_003372392
Blastopirellula_marina_ZP_01090148
Gemmata_obscuriglobus_ZP_02734407
Isosphaera_pallida_YP_004179901
Chthoniobacter_flavus_ZP_03132574
Vibrio_angustum_ZP_01237213
Cronobacter_sakazakii_YP_001436227
Escherichia_coli_AAC43134
Halomonas_elongata_YP_003899484
Pasteurella_multocida_NP_246690
Actinobacillus_pleuropneumoniae_YP_0016
Francisella_tularensis_YP_513138
Burkholderia_cenocepacia_YP_834375
Acidovorax_citrulli_YP_968974
Gloeobacter_violaceus_NP_926374
Anaplasma_marginale_YP_002563753
Babesia_bovis_XP_001611175
Schizosaccharomyces_pombe_NP_593219
Bordetella_bronchiseptica_NP_886652
Naegleria_gruberi_XP_002683169
Trichoplax_adhaerens_XP_002112583
Saccharomyces_cerevisiae_CAA96321
Lithospermum_erythrorhizon_BAB84122
Rhodospirillum_rubrum_YP_425663
Maricaulis_maris_YP_757472
Brevundimonas_subvibrioides_YP_00381784
Caulobacter_crescentus_YP_002518907
Rhodobacter_sphaeroides_YP_001044542
Xanthobacter_autotrophicus_YP_001416391
Ignicoccus_hospitalis_YP_001435047
Aeropyrum_pernix_NP_148031
Thermococcus_sp._YP_004885398
Thermococcus_gammatolerans_YP_002959596
Sulfolobus_solfataricus_AAK40480
Sulfolobus_acidocaldarius_YP_256904
Sulfolobus_tokodaii_NP_378201
Hydrogenobaculum_sp._YP_002121702
Desulfurispirillum_indicum_YP_004112212
Candidatus_Solibacter_YP_822588
Gemmatimonas_aurantiaca_YP_002761951
Pedobacter_sp._ZP_01884961
Deferribacter_desulfuricans_YP_00349557
Mitsuokella_multacida_ZP_05404624
Hydrogenobaculum_sp._YP_002122127
Syntrophobacter_fumaroxidans_YP_844270
Terriglobus_saanensis_YP_004181190
Sphingomonas_wittichii_YP_001261641
Xanthomonas_oryzae_YP_005630108
Chthoniobacter_flavus_ZP_03132114
Chlorobium_tepidum_NP_661078
Gallionella_capsiferriformans_YP_003848
Leptospira_interrogans_YP_000267
Herpetosiphon_aurantiacus_YP_001546007
Chloroflexus_aurantiacus_YP_001634142
Anaerolinea_thermophila_YP_004174219
Ktedonobacter_racemifer_ZP_06966970
Roseiflexus_castenholzii_YP_001434110
Geobacter_metallireducens_YP_384364
Porphyromonas_gingivalis_NP_904804
Leptotrichia_hofstadii_ZP_05902240
Sulfurovum_sp._YP_001358830
Sulfurimonas_denitrificans_YP_393729
Pedobacter_sp._ZP_01882493
0.873
0.911
0.64
0.995
0.789
0.307
0.345
0.724
0.838
0.846
0.687
0.781
0.93
0.991
0.286
0.565
0.855
0.97
0.782
0.989
0.891
0.806
0.867
0.117
0.681
0.825
0.659
0.634
0.725
0.902
0.929
0.948
0.861
0.978
0.556
0.398
0.833
0.611
0.888
0.999
0.409
1.0
0.987
0.914
0.953
0.838
0.898
0.902
0.058
0.077
0.532
0.99
0.999
0.631
0.463
0.938
0.416
0.788
0.897
0.774
0.932
0.907
0.804
0.806
0.834
0.057
0.841
0.217
0.872

SUPPLEMENTARY FIGURE 5: Phylogenetic tree of CDP alcohol phosphatidyltransferases probably related to serine transfer. This tree was reconstructed using 173 representative sequences and 132 conserved sites.

Suppl. Figure 5.

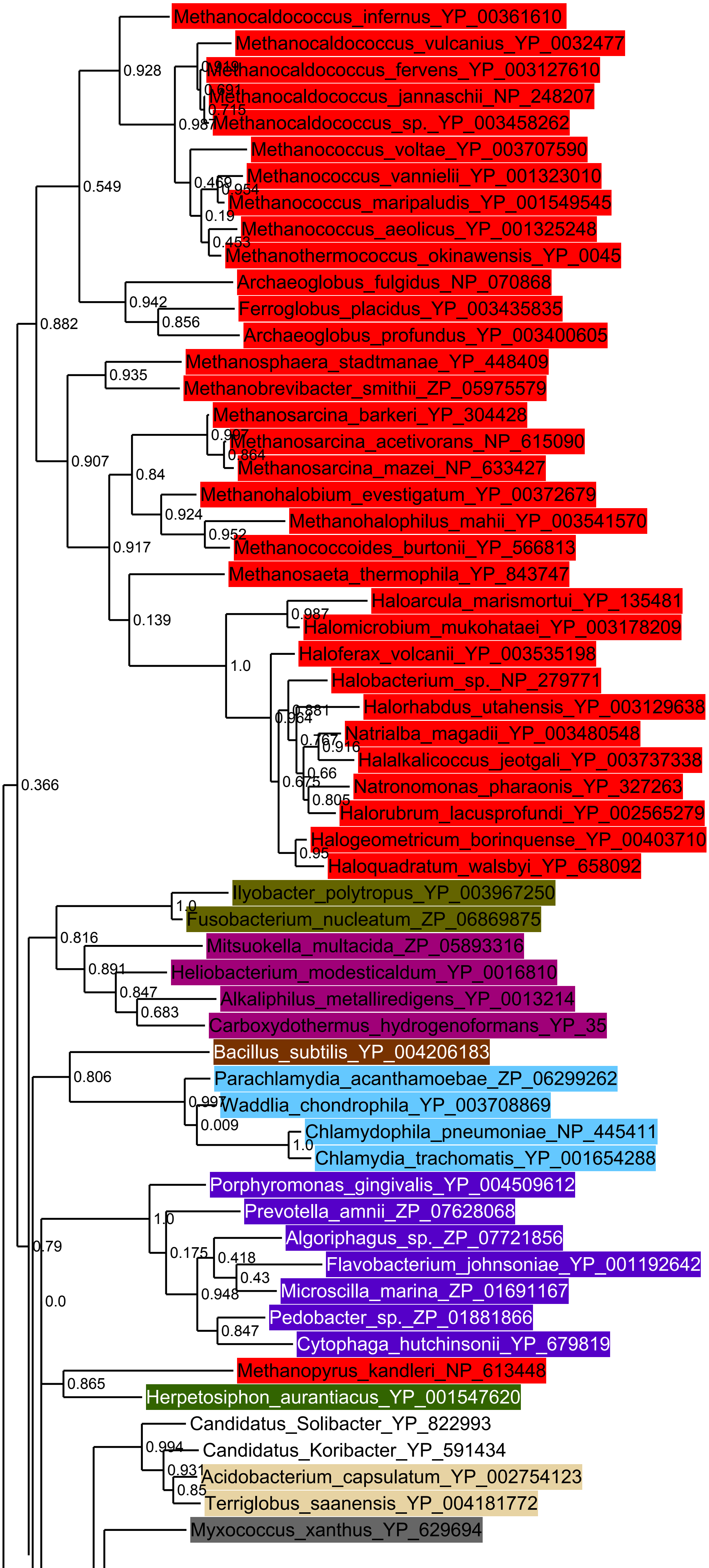

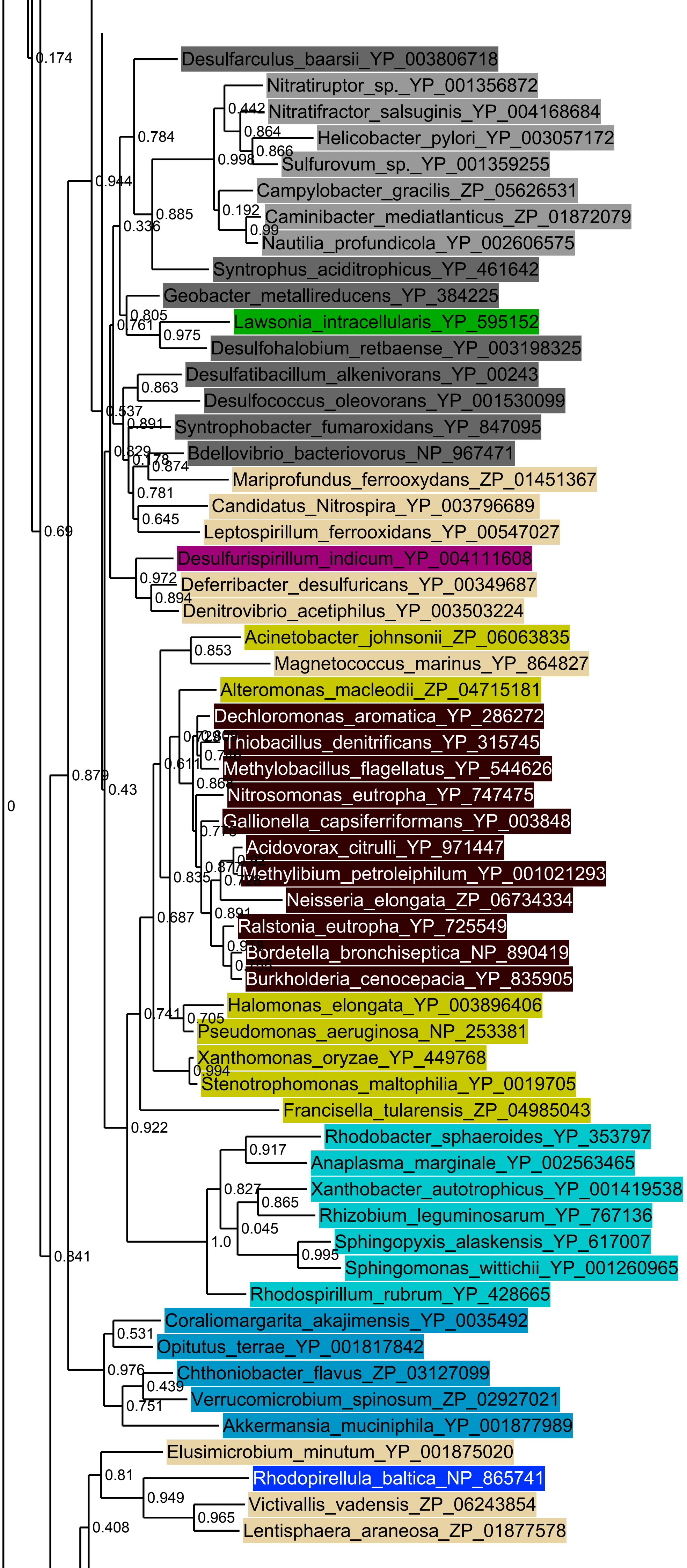

Desulfarculus_baarsii_YP_003806718
Nitratiruptor_sp._YP_001356872
Nitratifractor_salsuginis_YP_004168684
Helicobacter_pylori_YP_003057172
Sulfurovum_sp._YP_001359255
Campylobacter_gracilis_ZP_05626531
Caminibacter_mediatlanticus_ZP_01872079
Nautilia_profundicola_YP_002606575
Syntrophus_aciditrophicus_YP_461642
Geobacter_metallireducens_YP_384225
Lawsonia_intracellularis_YP_595152
Desulfohalobium_retbaense_YP_003198325
Desulfatibacillum_alkenivorans_YP_00243
Desulfococcus_oleovorans_YP_001530099
Syntrophobacter_fumaroxidans_YP_847095
Bdellovibrio_bacteriovorus_NP_967471
Mariprofundus_ferrooxydans_ZP_01451367
Candidatus_Nitrospira_YP_003796689
Leptospirillum_ferrooxidans_YP_00547027
Desulfurispirillum_indicum_YP_004111608
Deferribacter_desulfuricans_YP_00349687
Denitrovibrio_acetiphilus_YP_003503224
Acinetobacter_johnsonii_ZP_06063835
Magnetococcus_marinus_YP_864827
Alteromonas_macleodii_ZP_04715181
Dechloromonas_aromatica_YP_286272
Thiobacillus_denitrificans_YP_315745
Methylobacillus_flagellatus_YP_544626
Nitrosomonas_eutropha_YP_747475
Gallionella_capsiferriformans_YP_003848
Acidovorax_citrulli_YP_971447
Methylibium_petroleiphilum_YP_001021293
Neisseria_elongata_ZP_06734334
Ralstonia_eutropha_YP_725549
Bordetella_bronchiseptica_NP_890419
Burkholderia_cenocepacia_YP_835905
Halomonas_elongata_YP_003896406
Pseudomonas_aeruginosa_NP_253381
Xanthomonas_oryzae_YP_449768
Stenotrophomonas_maltophilia_YP_0019705
Francisella_tularensis_ZP_04985043
Rhodobacter_sphaeroides_YP_353797
Anaplasma_marginale_YP_002563465
Xanthobacter_autotrophicus_YP_001419538
Rhizobium_leguminosarum_YP_767136
Sphingopyxis_alaskensis_YP_617007
Sphingomonas_wittichii_YP_001260965
Rhodospirillum_rubrum_YP_428665
Coraliomargarita_akajimensis_YP_0035492
Opitutus_terrae_YP_001817842
Chthoniobacter_flavus_ZP_03127099
Verrucomicrobium_spinosum_ZP_02927021
Akkermansia_muciniphila_YP_001877989
Elusimicrobium_minutum_YP_001875020
Rhodopirellula_baltica_NP_865741
Victivallis_vadensis_ZP_06243854
Lentisphaera_araneosa_ZP_01877578
0.174
0.784
0.442
0.864
0.866
0.998
0.944
0.192
0.885
0.336
0.99
0.805
0.761
0.975
0.863
0.537
0.891
0.829
0.874
0.781
0.645
0.69
0.972
0.894
0.853
0.611
0.868
0.879
0.43
0.835
0.687
0.891
0.741
0.705
0.994
0.922
0.917
0.827
0.865
0.045
1.0
0.995
0.841
0
0.531
0.976
0.439
0.751
0.81
0.949
0.965
0.408

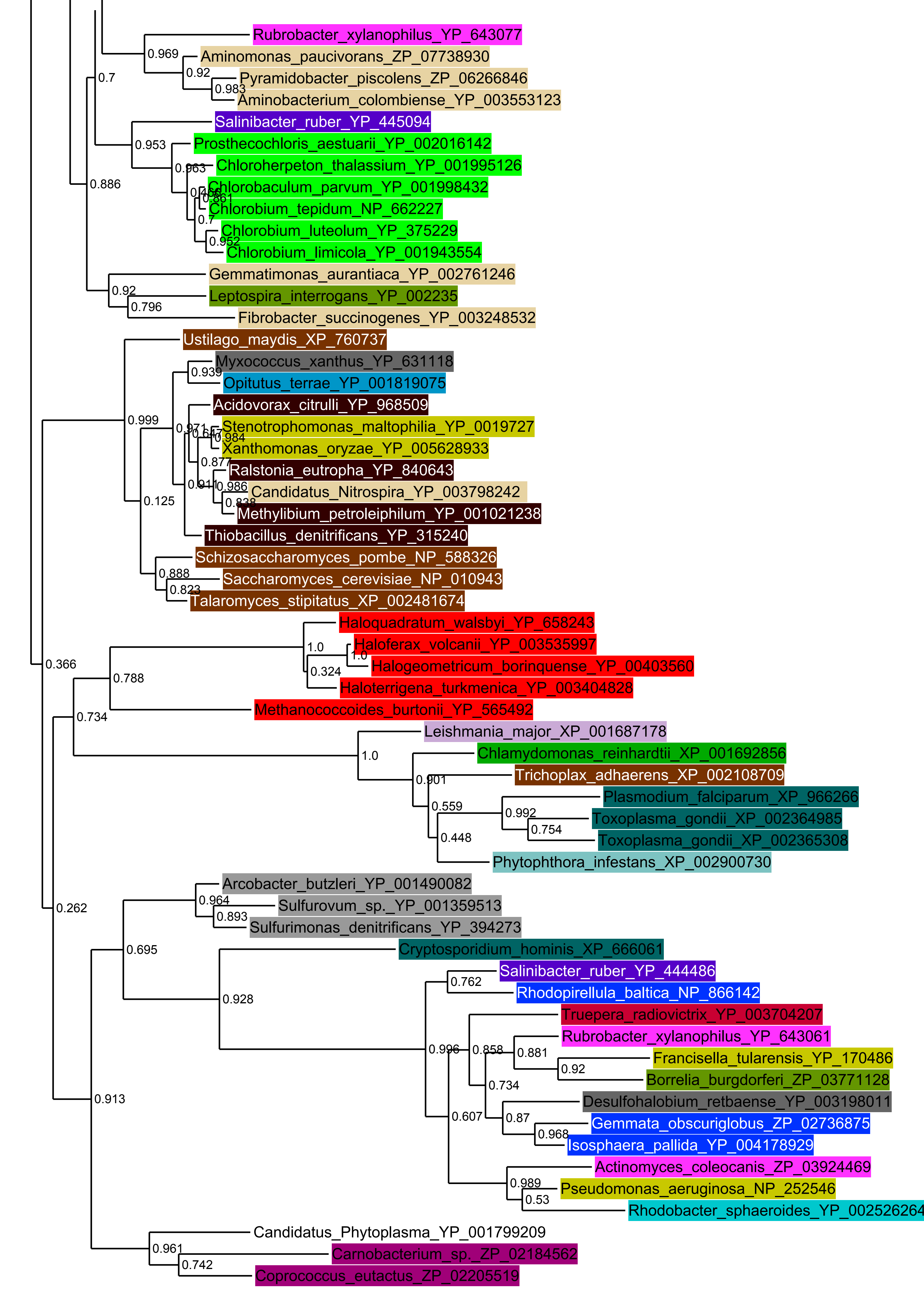

Rubrobacter_xylanophilus_YP_643077
0.969
Aminomonas_paucivorans_ZP_07738930
0.7
0.92
Pyramidobacter_piscolens_ZP_06266846
0.983
Aminobacterium_colombiense_YP_003553123
Salinibacter_ruber_YP_445094
0.953
Prosthecochloris_aestuarii_YP_002016142
0.963
Chloroherpeton_thalassium_YP_001995126
0.886
Chlorobaculum_parvum_YP_001998432
0.466
0.561
Chlorobium_tepidum_NP_662227
0.7
Chlorobium_luteolum_YP_375229
0.952
Chlorobium_limicola_YP_001943554
Gemmatimonas_aurantiaca_YP_002761246
0.92
Leptospira_interrogans_YP_002235
0.796
Fibrobacter_succinogenes_YP_003248532
Ustilago_maydis_XP_760737
Myxococcus_xanthus_YP_631118
0.939
Opitutus_terrae_YP_001819075
Acidovorax_citrulli_YP_968509
0.999
0.971
Stenotrophomonas_maltophilia_YP_0019727
0.647
0.984
Xanthomonas_oryzae_YP_005628933
0.877
Ralstonia_eutropha_YP_840643
0.911
0.986
Candidatus_Nitrospira_YP_003798242
0.125
0.838
Methylibium_petroleiphilum_YP_001021238
Thiobacillus_denitrificans_YP_315240
Schizosaccharomyces_pombe_NP_588326
0.888
Saccharomyces_cerevisiae_NP_010943
0.823
Talaromyces_stipitatus_XP_002481674
Haloquadratum_walsbyi_YP_658243
1.0
Haloferax_volcanii_YP_003535997
1.0
0.366
Halogeometricum_borinquense_YP_00403560
0.324
0.788
Haloterrigena_turkmenica_YP_003404828
0.734
Methanococcoides_burtonii_YP_565492
Leishmania_major_XP_001687178
1.0
Chlamydomonas_reinhardtii_XP_001692856
Trichoplax_adhaerens_XP_002108709
0.901
Plasmodium_falciparum_XP_966266
0.559
0.992
Toxoplasma_gondii_XP_002364985
0.754
0.448
Toxoplasma_gondii_XP_002365308
Phytophthora_infestans_XP_002900730
Arcobacter_butzleri_YP_001490082
0.964
Sulfurovum_sp._YP_001359513
0.262
0.893
Sulfurimonas_denitrificans_YP_394273
Cryptosporidium_hominis_XP_666061
0.695
Salinibacter_ruber_YP_444486
0.762
0.928
Rhodopirellula_baltica_NP_866142
Truepera_radiovictrix_YP_003704207
Rubrobacter_xylanophilus_YP_643061
0.996
0.858
0.881
Francisella_tularensis_YP_170486
0.92
Borrelia_burgdorferi_ZP_03771128
0.734
0.913
Desulfohalobium_retbaense_YP_003198011
0.607
0.87
Gemmata_obscuriglobus_ZP_02736875
0.968
Isosphaera_pallida_YP_004178929
Actinomyces_coleocanis_ZP_03924469
0.989
Pseudomonas_aeruginosa_NP_252546
0.53
Rhodobacter_sphaeroides_YP_002526264
Candidatus_Phytoplasma_YP_001799209
0.961
Carnobacterium_sp._ZP_02184562
0.742
Coprococcus_eutactus_ZP_02205519

SUPPLEMENTARY FIGURE 6: Phylogenetic tree of CDP alcohol phosphatidyltransferases probably related to *myo*-inositol transfer. This tree was reconstructed using 160 representative sequences and 159 conserved sites.

Suppl. Figure 6.

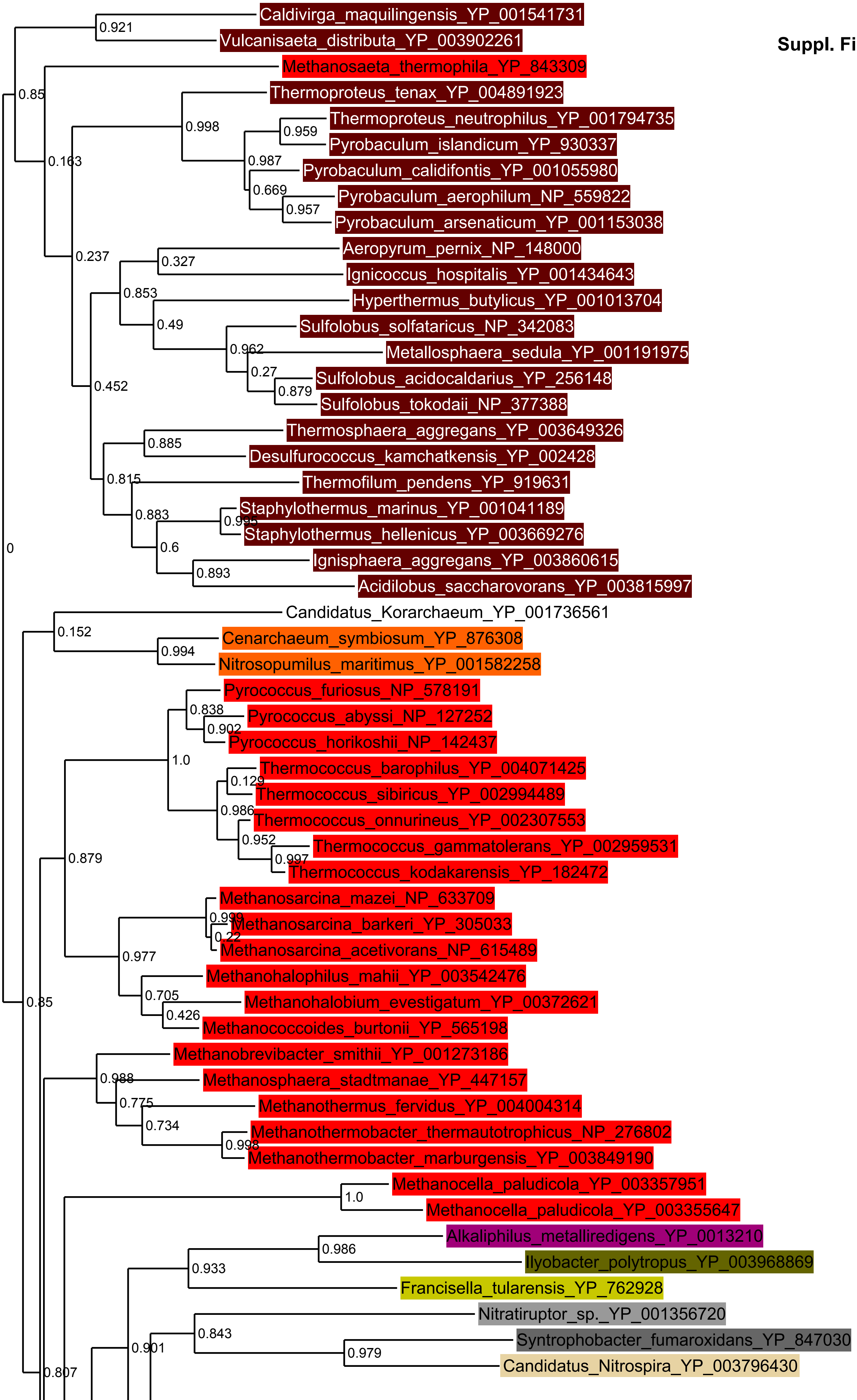

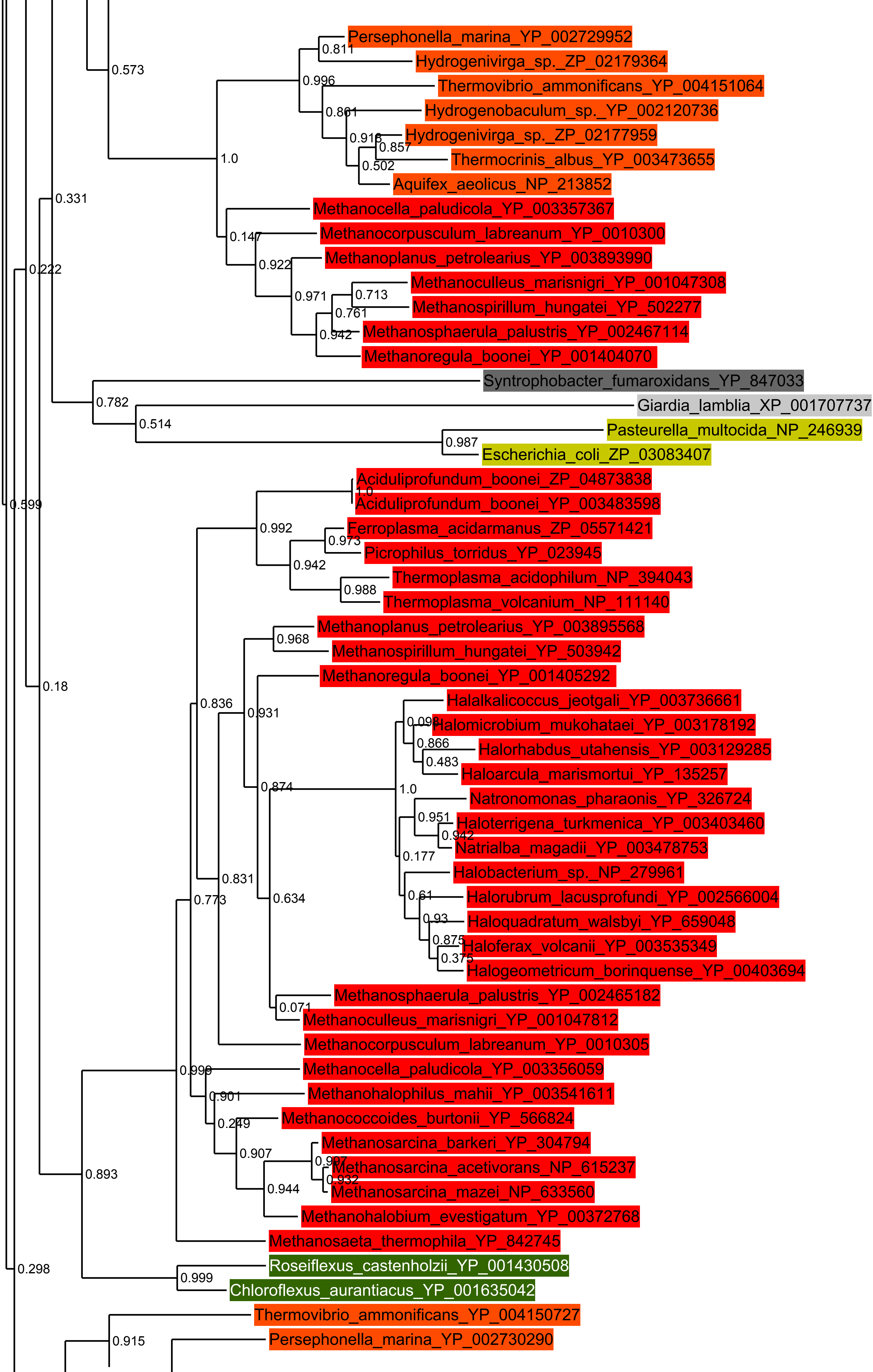

Persephonella_marina_YP_002729952
0.811
Hydrogenivirga_sp._ZP_02179364
0.573
0.996
Thermovibrio_ammonificans_YP_004151064
0.861
Hydrogenobaculum_sp._YP_002120736
0.918
Hydrogenivirga_sp._ZP_02177959
0.857
1.0
Thermocrinis_albus_YP_003473655
0.502
Aquifex_aeolicus_NP_213852
0.331
Methanocella_paludicola_YP_003357367
0.147
Methanocorpusculum_labreanum_YP_0010300
0.922
Methanoplanus_petrolearius_YP_003893990
0.222
Methanoculleus_marisnigri_YP_001047308
0.971
0.713
Methanospirillum_hungatei_YP_502277
0.761
0.942
Methanosphaerula_palustris_YP_002467114
Methanoregula_boonei_YP_001404070
Syntrophobacter_fumaroxidans_YP_847033
0.782
Giardia_lamblia_XP_001707737
0.514
Pasteurella_multocida_NP_246939
0.987
Escherichia_coli_ZP_03083407
Aciduliprofundum_boonei_ZP_04873838
1.0
0.599
Aciduliprofundum_boonei_YP_003483598
0.992
Ferroplasma_acidarmanus_ZP_05571421
0.973
Picrophilus_torridus_YP_023945
0.942
Thermoplasma_acidophilum_NP_394043
0.988
Thermoplasma_volcanium_NP_111140
Methanoplanus_petrolearius_YP_003895568
0.968
Methanospirillum_hungatei_YP_503942
Methanoregula_boonei_YP_001405292
0.18
Halalkalicoccus_jeotgali_YP_003736661
0.836
0.931
0.098
Halomicrobium_mukohataei_YP_003178192
0.866
Halorhabdus_utahensis_YP_003129285
0.483
Haloarcula_marismortui_YP_135257
0.874
1.0
Natronomonas_pharaonis_YP_326724
0.951
Haloterrigena_turkmenica_YP_003403460
0.942
Natrialba_magadii_YP_003478753
0.177
Halobacterium_sp._NP_279961
0.831
0.61
Halorubrum_lacusprofundi_YP_002566004
0.773
0.634
0.93
Haloquadratum_walsbyi_YP_659048
0.875
Haloferax_volcanii_YP_003535349
0.375
Halogeometricum_borinquense_YP_00403694
Methanosphaerula_palustris_YP_002465182
0.071
Methanoculleus_marisnigri_YP_001047812
Methanocorpusculum_labreanum_YP_0010305
0.999
Methanocella_paludicola_YP_003356059
0.901
Methanohalophilus_mahii_YP_003541611
0.249
Methanococcoides_burtonii_YP_566824
Methanosarcina_barkeri_YP_304794
0.907
0.997
Methanosarcina_acetivorans_NP_615237
0.893
0.932
0.944
Methanosarcina_mazei_NP_633560
Methanohalobium_evestigatum_YP_00372768
Methanosaeta_thermophila_YP_842745
0.298
Roseiflexus_castenholzii_YP_001430508
0.999
Chloroflexus_aurantiacus_YP_001635042
Thermovibrio_ammonificans_YP_004150727
0.915
Persephonella_marina_YP_002730290

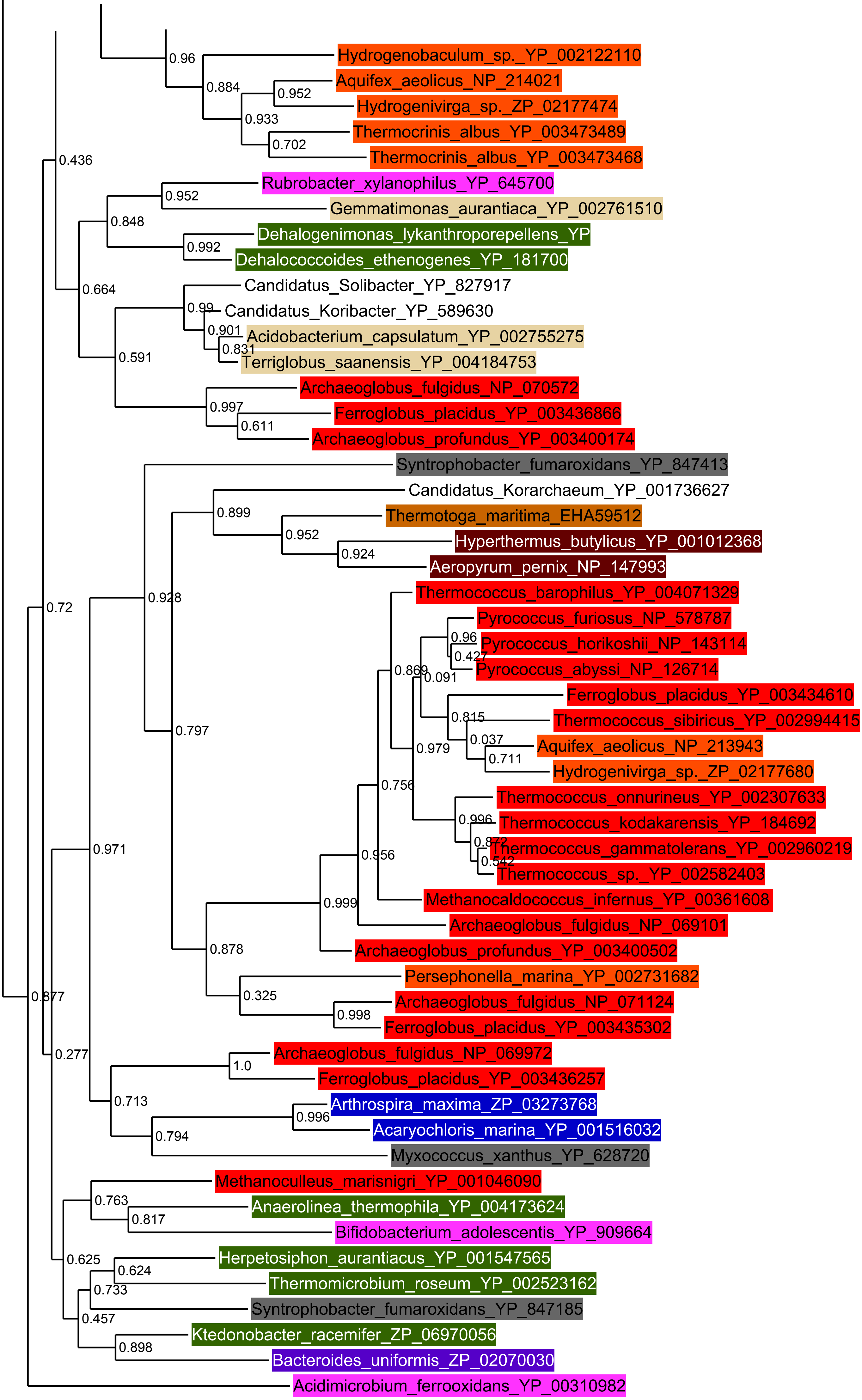
Hydrogenobaculum_sp._YP_002122110
Aquifex_aeolicus_NP_214021
Hydrogenivirga_sp._ZP_02177474
Thermocrinis_albus_YP_003473489
Thermocrinis_albus_YP_003473468
Rubrobacter_xylanophilus_YP_645700
Gemmatimonas_aurantiaca_YP_002761510
Dehalogenimonas_lykanthroporepellens_YP
Dehalococcoides_ethenogenes_YP_181700
Candidatus_Solibacter_YP_827917
Candidatus_Koribacter_YP_589630
Acidobacterium_capsulatum_YP_002755275
Terriglobus_saanensis_YP_004184753
Archaeoglobus_fulgidus_NP_070572
Ferroglobus_placidus_YP_003436866
Archaeoglobus_profundus_YP_003400174
Syntrophobacter_fumaroxidans_YP_847413
Candidatus_Korarchaeum_YP_001736627
Thermotoga_maritima_EHA59512
Hyperthermus_butylicus_YP_001012368
Aeropyrum_pernix_NP_147993
Thermococcus_barophilus_YP_004071329
Pyrococcus_furiosus_NP_578787
Pyrococcus_horikoshii_NP_143114
Pyrococcus_abyssi_NP_126714
Ferroglobus_placidus_YP_003434610
Thermococcus_sibiricus_YP_002994415
Aquifex_aeolicus_NP_213943
Hydrogenivirga_sp._ZP_02177680
Thermococcus_onnurineus_YP_002307633
Thermococcus_kodakarensis_YP_184692
Thermococcus_gammatolerans_YP_002960219
Thermococcus_sp._YP_002582403
Methanocaldococcus_infernus_YP_00361608
Archaeoglobus_fulgidus_NP_069101
Archaeoglobus_profundus_YP_003400502
Persephonella_marina_YP_002731682
Archaeoglobus_fulgidus_NP_071124
Ferroglobus_placidus_YP_003435302
Archaeoglobus_fulgidus_NP_069972
Ferroglobus_placidus_YP_003436257
Arthrospira_maxima_ZP_03273768
Acaryochloris_marina_YP_001516032
Myxococcus_xanthus_YP_628720
Methanoculleus_marisnigri_YP_001046090
Anaerolinea_thermophila_YP_004173624
Bifidobacterium_adolescentis_YP_909664
Herpetosiphon_aurantiacus_YP_001547565
Thermomicrobium_roseum_YP_002523162
Syntrophobacter_fumaroxidans_YP_847185
Ktedonobacter_racemifer_ZP_06970056
Bacteroides_uniformis_ZP_02070030
Acidimicrobium_ferrooxidans_YP_00310982
0.96
0.884
0.952
0.933
0.702
0.436
0.952
0.848
0.992
0.664
0.99
0.901
0.831
0.591
0.997
0.611
0.72
0.899
0.952
0.924
0.928
0.96
0.427
0.869
0.091
0.815
0.037
0.711
0.797
0.979
0.756
0.996
0.872
0.542
0.956
0.999
0.971
0.878
0.325
0.998
0.877
0.277
1.0
0.713
0.996
0.794
0.763
0.817
0.625
0.624
0.733
0.457
0.898

SUPPLEMENTARY FIGURE 7: Phylogenetic tree of geranylgeranyl reductases (GGR). This tree was reconstructed using 317 representative sequences and 266 conserved sites.

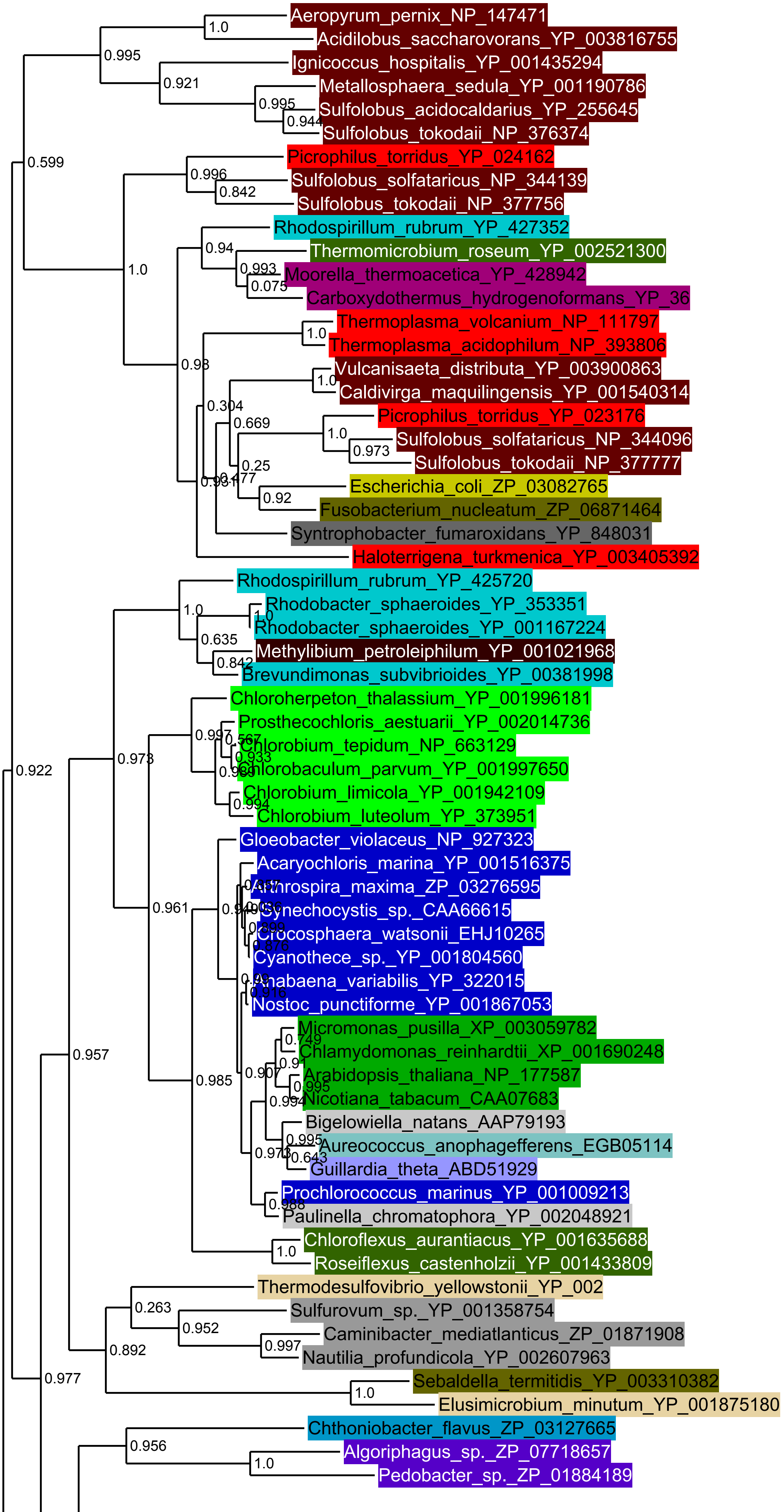

Suppl. Figure 7.

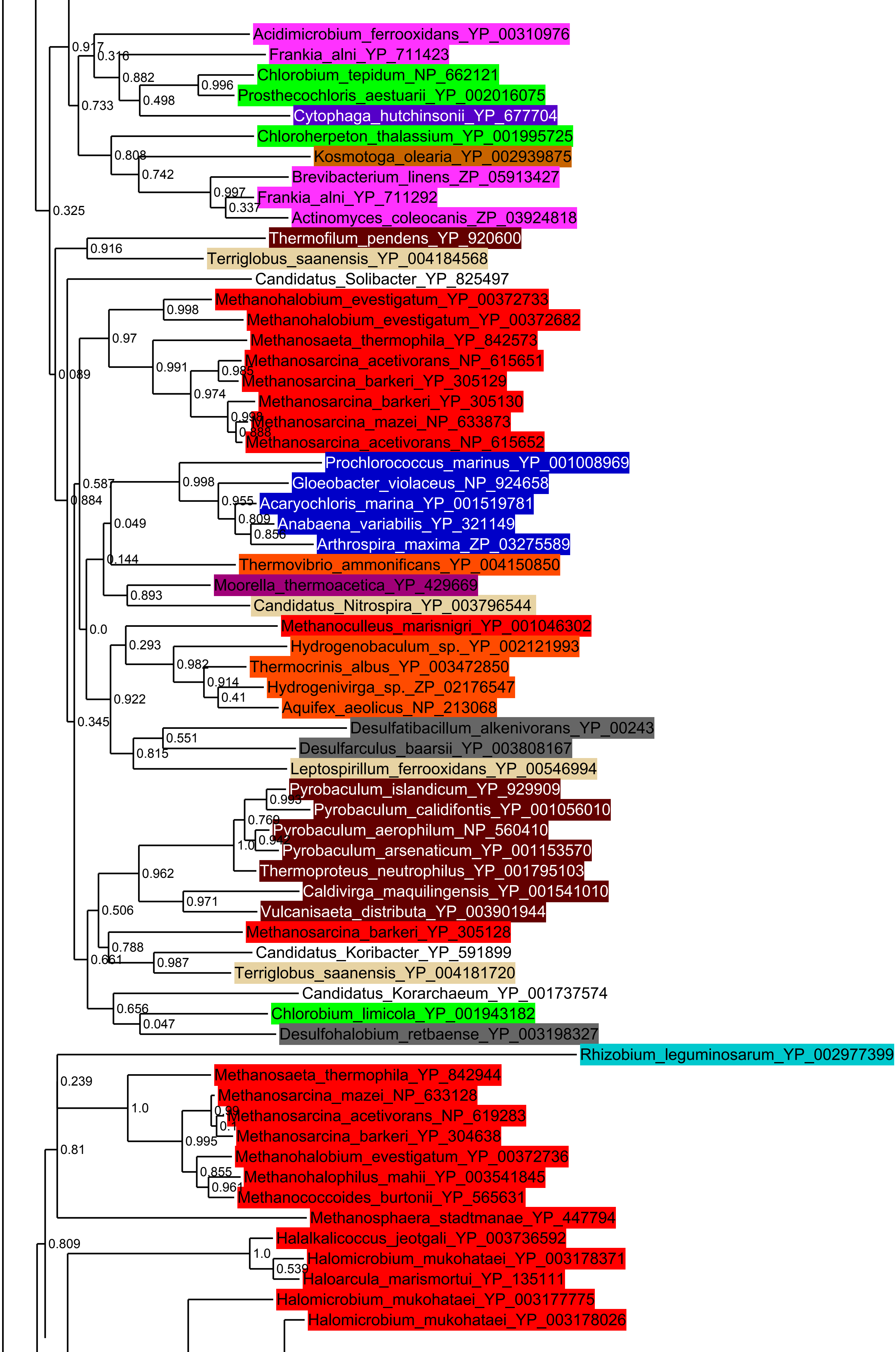

Acidimicrobium_ferrooxidans_YP_00310976
0.917
0.316
Frankia_alni_YP_711423
0.882
Chlorobium_tepidum_NP_662121
0.996
0.733
0.498
Prosthecochloris_aestuarii_YP_002016075
Cytophaga_hutchinsonii_YP_677704
Chloroherpeton_thalassium_YP_001995725
0.808
Kosmotoga_olearia_YP_002939875
0.742
Brevibacterium_linens_ZP_05913427
0.997
Frankia_alni_YP_711292
0.325
0.337
Actinomyces_coleocanis_ZP_03924818
Thermofilum_pendens_YP_920600
0.916
Terriglobus_saanensis_YP_004184568
Candidatus_Solibacter_YP_825497
Methanohalobium_evestigatum_YP_00372733
0.998
Methanohalobium_evestigatum_YP_00372682
0.97
Methanosaeta_thermophila_YP_842573
Methanosarcina_acetivorans_NP_615651
0.991
0.089
Methanosarcina_barkeri_YP_305129
0.974
Methanosarcina_barkeri_YP_305130
Methanosarcina_mazei_NP_633873
0.888
Methanosarcina_acetivorans_NP_615652
Prochlorococcus_marinus_YP_001008969
0.587
0.998
Gloeobacter_violaceus_NP_924658
0.884
0.955
Acaryochloris_marina_YP_001519781
0.049
0.809
Anabaena_variabilis_YP_321149
0.856
Arthrospira_maxima_ZP_03275589
0.144
Thermovibrio_ammonificans_YP_004150850
Moorella_thermoacetica_YP_429669
0.893
Candidatus_Nitrospira_YP_003796544
0.0
Methanoculleus_marisnigri_YP_001046302
0.293
Hydrogenobaculum_sp._YP_002121993
0.982
Thermocrinis_albus_YP_003472850
0.914
Hydrogenivirga_sp._ZP_02176547
0.922
0.41
Aquifex_aeolicus_NP_213068
0.345
Desulfatibacillum_alkenivorans_YP_00243
0.551
Desulfarculus_baarsii_YP_003808167
0.815
Leptospirillum_ferrooxidans_YP_00546994
Pyrobaculum_islandicum_YP_929909
0.993
Pyrobaculum_calidifontis_YP_001056010
0.769
Pyrobaculum_aerophilum_NP_560410
1.0
Pyrobaculum_arsenaticum_YP_001153570
0.962
Thermoproteus_neutrophilus_YP_001795103
Caldivirga_maquilingensis_YP_001541010
0.971
0.506
Vulcanisaeta_distributa_YP_003901944
Methanosarcina_barkeri_YP_305128
0.788
Candidatus_Koribacter_YP_591899
0.661
0.987
Terriglobus_saanensis_YP_004181720
Candidatus_Korarchaeum_YP_001737574
0.656
Chlorobium_limicola_YP_001943182
0.047
Desulfohalobium_retbaense_YP_003198327
Rhizobium_leguminosarum_YP_002977399
0.239
Methanosaeta_thermophila_YP_842944
Methanosarcina_mazei_NP_633128
1.0
0.99
Methanosarcina_acetivorans_NP_619283
Methanosarcina_barkeri_YP_304638
0.81
0.995
Methanohalobium_evestigatum_YP_00372736
0.855
Methanohalophilus_mahii_YP_003541845
0.961
Methanococcoides_burtonii_YP_565631
Methanosphaera_stadtmanae_YP_447794
Halalkalicoccus_jeotgali_YP_003736592
0.809
1.0
Halomicrobium_mukohataei_YP_003178371
0.539
Haloarcula_marismortui_YP_135111
Halomicrobium_mukohataei_YP_003177775
Halomicrobium_mukohataei_YP_003178026

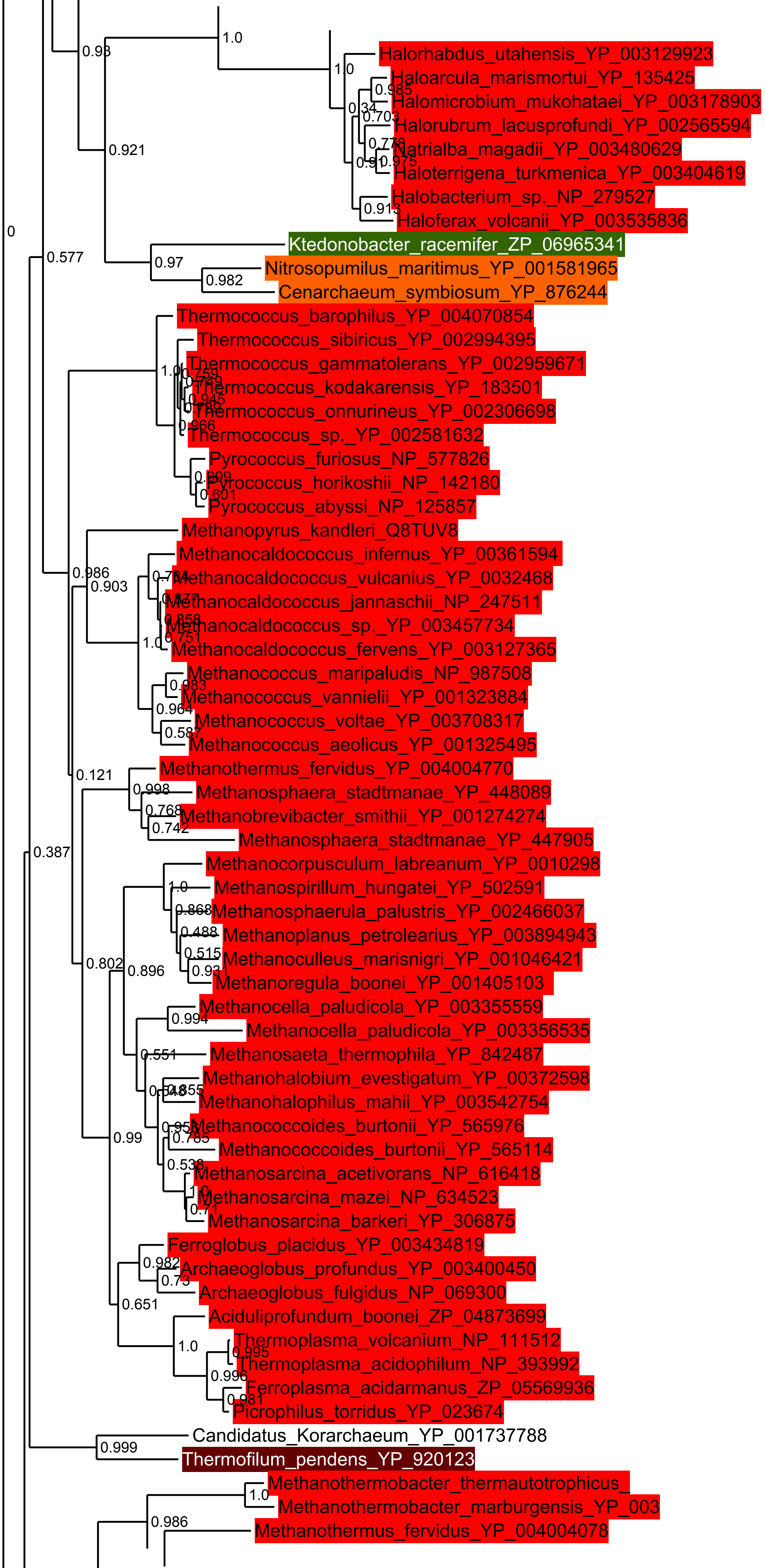

Halorhabdus_utahensis_YP_003129923
Haloarcula_marismortui_YP_135425
Halomicrobium_mukohataei_YP_003178903
Halorubrum_lacusprofundi_YP_002565594
Natrialba_magadii_YP_003480629
Haloterrigena_turkmenica_YP_003404619
Halobacterium_sp._NP_279527
Haloferax_volcanii_YP_003535836
Ktedonobacter_racemifer_ZP_06965341
Nitrosopumilus_maritimus_YP_001581965
Cenarchaeum_symbiosum_YP_876244
Thermococcus_barophilus_YP_004070854
Thermococcus_sibiricus_YP_002994395
Thermococcus_gammatolerans_YP_002959671
Thermococcus_kodakarensis_YP_183501
Thermococcus_onnurineus_YP_002306698
Thermococcus_sp._YP_002581632
Pyrococcus_furiosus_NP_577826
Pyrococcus_horikoshii_NP_142180
Pyrococcus_abyssi_NP_125857
Methanopyrus_kandleri_Q8TUV8
Methanocaldococcus_infernus_YP_00361594
Methanocaldococcus_vulcanius_YP_0032468
Methanocaldococcus_jannaschii_NP_247511
Methanocaldococcus_sp._YP_003457734
Methanocaldococcus_fervens_YP_003127365
Methanococcus_maripaludis_NP_987508
Methanococcus_vannielii_YP_001323884
Methanococcus_voltae_YP_003708317
Methanococcus_aeolicus_YP_001325495
Methanothermus_fervidus_YP_004004770
Methanosphaera_stadtmanae_YP_448089
Methanobrevibacter_smithii_YP_001274274
Methanosphaera_stadtmanae_YP_447905
Methanocorpusculum_labreanum_YP_0010298
Methanospirillum_hungatei_YP_502591
Methanosphaerula_palustris_YP_002466037
Methanoplanus_petrolearius_YP_003894943
Methanoculleus_marisnigri_YP_001046421
Methanoregula_boonei_YP_001405103
Methanocella_paludicola_YP_003355559
Methanocella_paludicola_YP_003356535
Methanosaeta_thermophila_YP_842487
Methanohalobium_evestigatum_YP_00372598
Methanohalophilus_mahii_YP_003542754
Methanococcoides_burtonii_YP_565976
Methanococcoides_burtonii_YP_565114
Methanosarcina_acetivorans_NP_616418
Methanosarcina_mazei_NP_634523
Methanosarcina_barkeri_YP_306875
Ferroglobus_placidus_YP_003434819
Archaeoglobus_profundus_YP_003400450
Archaeoglobus_fulgidus_NP_069300
Aciduliprofundum_boonei_ZP_04873699
Thermoplasma_volcanium_NP_111512
Thermoplasma_acidophilum_NP_393992
Ferroplasma_acidarmanus_ZP_05569936
Picrophilus_torridus_YP_023674
Candidatus_Korarchaeum_YP_001737788
Thermofilum_pendens_YP_920123
Methanothermobacter_thermautotrophicus_
Methanothermobacter_marburgensis_YP_003
Methanothermus_fervidus_YP_004004078

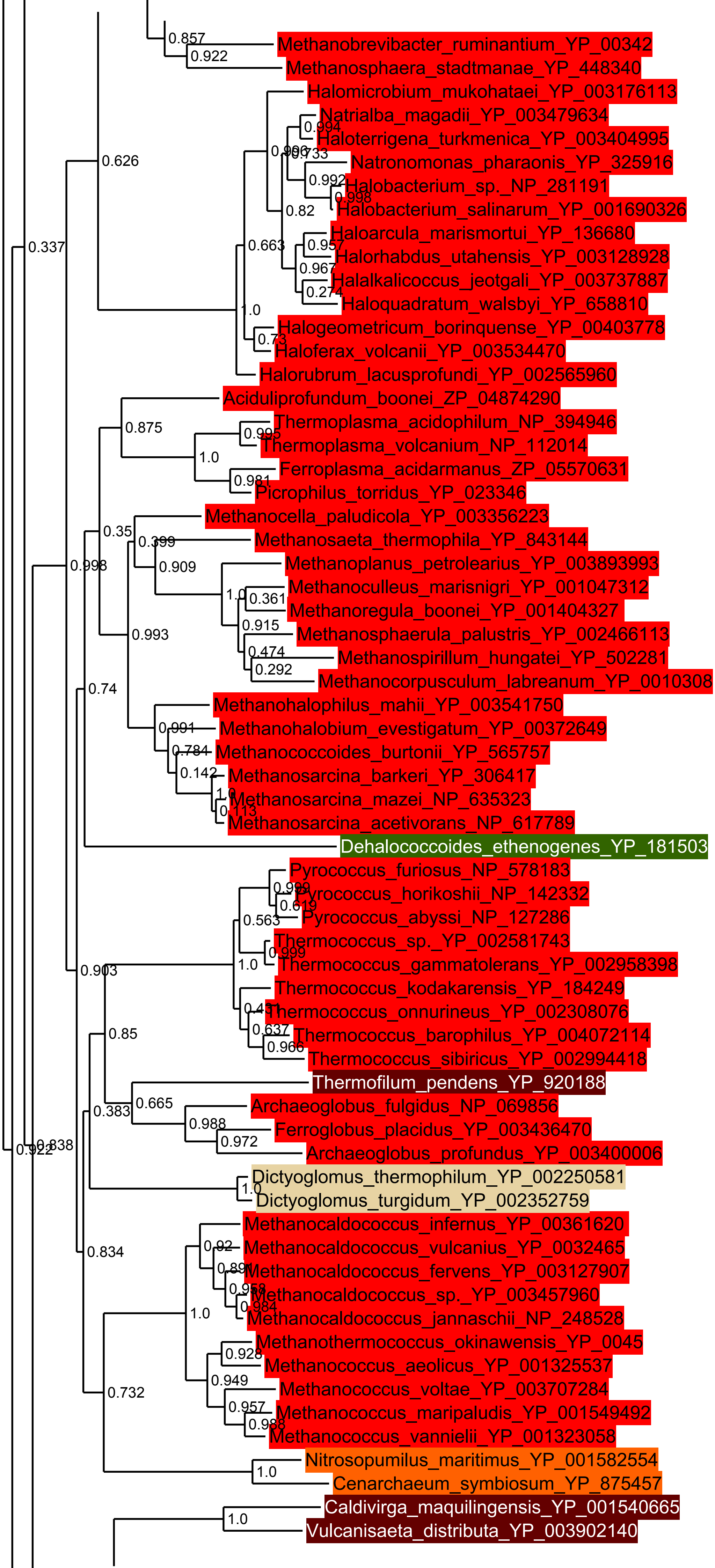

Methanobrevibacter_ruminantium_YP_00342
Methanosphaera_stadtmanae_YP_448340
Halomicrobium_mukohataei_YP_003176113
Natrialba_magadii_YP_003479634
Haloterrigena_turkmenica_YP_003404995
Natronomonas_pharaonis_YP_325916
Halobacterium_sp._NP_281191
Halobacterium_salinarum_YP_001690326
Haloarcula_marismortui_YP_136680
Halorhabdus_utahensis_YP_003128928
Halalkalicoccus_jeotgali_YP_003737887
Haloquadratum_walsbyi_YP_658810
Halogeometricum_borinquense_YP_00403778
Haloferax_volcanii_YP_003534470
Halorubrum_lacusprofundi_YP_002565960
Aciduliprofundum_boonei_ZP_04874290
Thermoplasma_acidophilum_NP_394946
Thermoplasma_volcanium_NP_112014
Ferroplasma_acidarmanus_ZP_05570631
Picrophilus_torridus_YP_023346
Methanocella_paludicola_YP_003356223
Methanosaeta_thermophila_YP_843144
Methanoplanus_petrolearius_YP_003893993
Methanoculleus_marisnigri_YP_001047312
Methanoregula_boonei_YP_001404327
Methanosphaerula_palustris_YP_002466113
Methanospirillum_hungatei_YP_502281
Methanocorpusculum_labreanum_YP_0010308
Methanohalophilus_mahii_YP_003541750
Methanohalobium_evestigatum_YP_00372649
Methanococcoides_burtonii_YP_565757
Methanosarcina_barkeri_YP_306417
Methanosarcina_mazei_NP_635323
Methanosarcina_acetivorans_NP_617789
Dehalococcoides_ethenogenes_YP_181503
Pyrococcus_furiosus_NP_578183
Pyrococcus_horikoshii_NP_142332
Pyrococcus_abyssi_NP_127286
Thermococcus_sp._YP_002581743
Thermococcus_gammatolerans_YP_002958398
Thermococcus_kodakarensis_YP_184249
Thermococcus_onnurineus_YP_002308076
Thermococcus_barophilus_YP_004072114
Thermococcus_sibiricus_YP_002994418
Thermofilum_pendens_YP_920188
Archaeoglobus_fulgidus_NP_069856
Ferroglobus_placidus_YP_003436470
Archaeoglobus_profundus_YP_003400006
Dictyoglomus_thermophilum_YP_002250581
Dictyoglomus_turgidum_YP_002352759
Methanocaldococcus_infernus_YP_00361620
Methanocaldococcus_vulcanius_YP_0032465
Methanocaldococcus_fervens_YP_003127907
Methanocaldococcus_sp._YP_003457960
Methanocaldococcus_jannaschii_NP_248528
Methanothermococcus_okinawensis_YP_0045
Methanococcus_aeolicus_YP_001325537
Methanococcus_voltae_YP_003707284
Methanococcus_maripaludis_YP_001549492
Methanococcus_vannielii_YP_001323058
Nitrosopumilus_maritimus_YP_001582554
Cenarchaeum_symbiosum_YP_875457
Caldivirga_maquilingensis_YP_001540665
Vulcanisaeta_distributa_YP_003902140
0.857
0.922
0.626
0.337
0.994
0.996
0.733
0.992
0.998
0.82
0.663
0.957
0.967
0.274
1.0
0.73
0.875
0.995
1.0
0.981
0.35
0.399
0.998
0.909
1.0
0.361
0.993
0.915
0.474
0.292
0.74
0.991
0.784
0.142
1.0
0.113
0.999
0.619
0.563
0.999
0.903
1.0
0.431
0.637
0.966
0.85
0.665
0.383
0.988
0.972
0.922
0.838
1.0
0.92
0.89
0.958
0.984
0.834
1.0
0.928
0.949
0.732
0.957
0.988
1.0
1.0

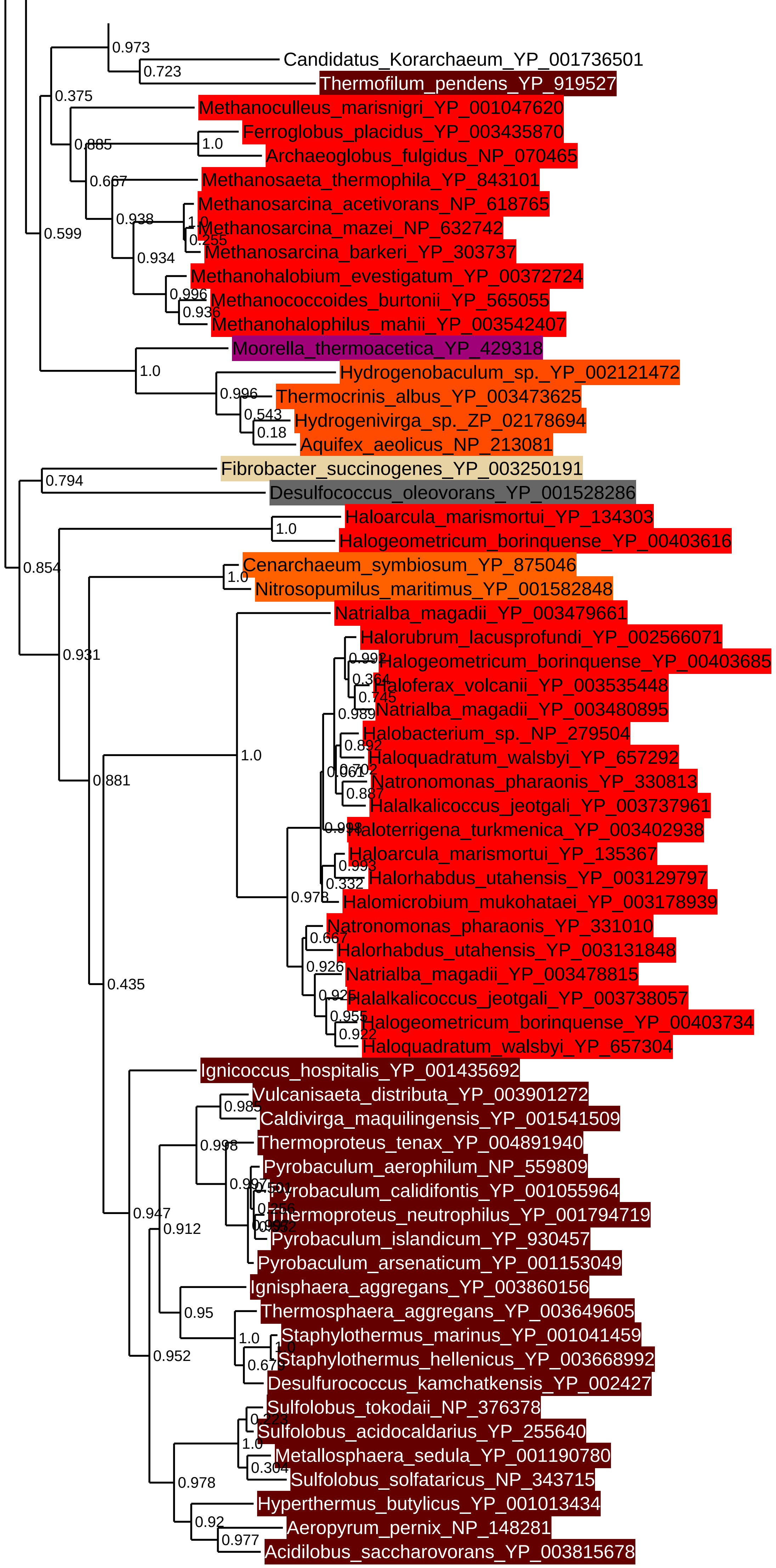

Candidatus_Korarchaeum_YP_001736501
Thermofilum_pendens_YP_919527
Methanoculleus_marisnigri_YP_001047620
Ferroglobus_placidus_YP_003435870
Archaeoglobus_fulgidus_NP_070465
Methanosaeta_thermophila_YP_843101
Methanosarcina_acetivorans_NP_618765
Methanosarcina_mazei_NP_632742
Methanosarcina_barkeri_YP_303737
Methanohalobium_evestigatum_YP_00372724
Methanococcoides_burtonii_YP_565055
Methanohalophilus_mahii_YP_003542407
Moorella_thermoacetica_YP_429318
Hydrogenobaculum_sp._YP_002121472
Thermocrinis_albus_YP_003473625
Hydrogenivirga_sp._ZP_02178694
Aquifex_aeolicus_NP_213081
Fibrobacter_succinogenes_YP_003250191
Desulfococcus_oleovorans_YP_001528286
Haloarcula_marismortui_YP_134303
Halogeometricum_borinquense_YP_00403616
Cenarchaeum_symbiosum_YP_875046
Nitrosopumilus_maritimus_YP_001582848
Natrialba_magadii_YP_003479661
Halorubrum_lacusprofundi_YP_002566071
Halogeometricum_borinquense_YP_00403685
Haloferax_volcanii_YP_003535448
Natrialba_magadii_YP_003480895
Halobacterium_sp._NP_279504
Haloquadratum_walsbyi_YP_657292
Natronomonas_pharaonis_YP_330813
Halalkalicoccus_jeotgali_YP_003737961
Haloterrigena_turkmenica_YP_003402938
Haloarcula_marismortui_YP_135367
Halorhabdus_utahensis_YP_003129797
Halomicrobium_mukohataei_YP_003178939
Natronomonas_pharaonis_YP_331010
Halorhabdus_utahensis_YP_003131848
Natrialba_magadii_YP_003478815
Halalkalicoccus_jeotgali_YP_003738057
Halogeometricum_borinquense_YP_00403734
Haloquadratum_walsbyi_YP_657304
Ignicoccus_hospitalis_YP_001435692
Vulcanisaeta_distributa_YP_003901272
Caldivirga_maquilingensis_YP_001541509
Thermoproteus_tenax_YP_004891940
Pyrobaculum_aerophilum_NP_559809
Pyrobaculum_calidifontis_YP_001055964
Thermoproteus_neutrophilus_YP_001794719
Pyrobaculum_islandicum_YP_930457
Pyrobaculum_arsenaticum_YP_001153049
Ignisphaera_aggregans_YP_003860156
Thermosphaera_aggregans_YP_003649605
Staphylothermus_marinus_YP_001041459
Staphylothermus_hellenicus_YP_003668992
Desulfurococcus_kamchatkensis_YP_002427
Sulfolobus_tokodaii_NP_376378
Sulfolobus_acidocaldarius_YP_255640
Metallosphaera_sedula_YP_001190780
Sulfolobus_solfataricus_NP_343715
Hyperthermus_butylicus_YP_001013434
Aeropyrum_pernix_NP_148281
Acidilobus_saccharovorans_YP_003815678

SUPPLEMENTARY FIGURE 8: Phylogenetic tree of CDP diglyceryde synthases (CdsA). This tree was reconstructed using 133 representative sequences and 87 conserved sites.

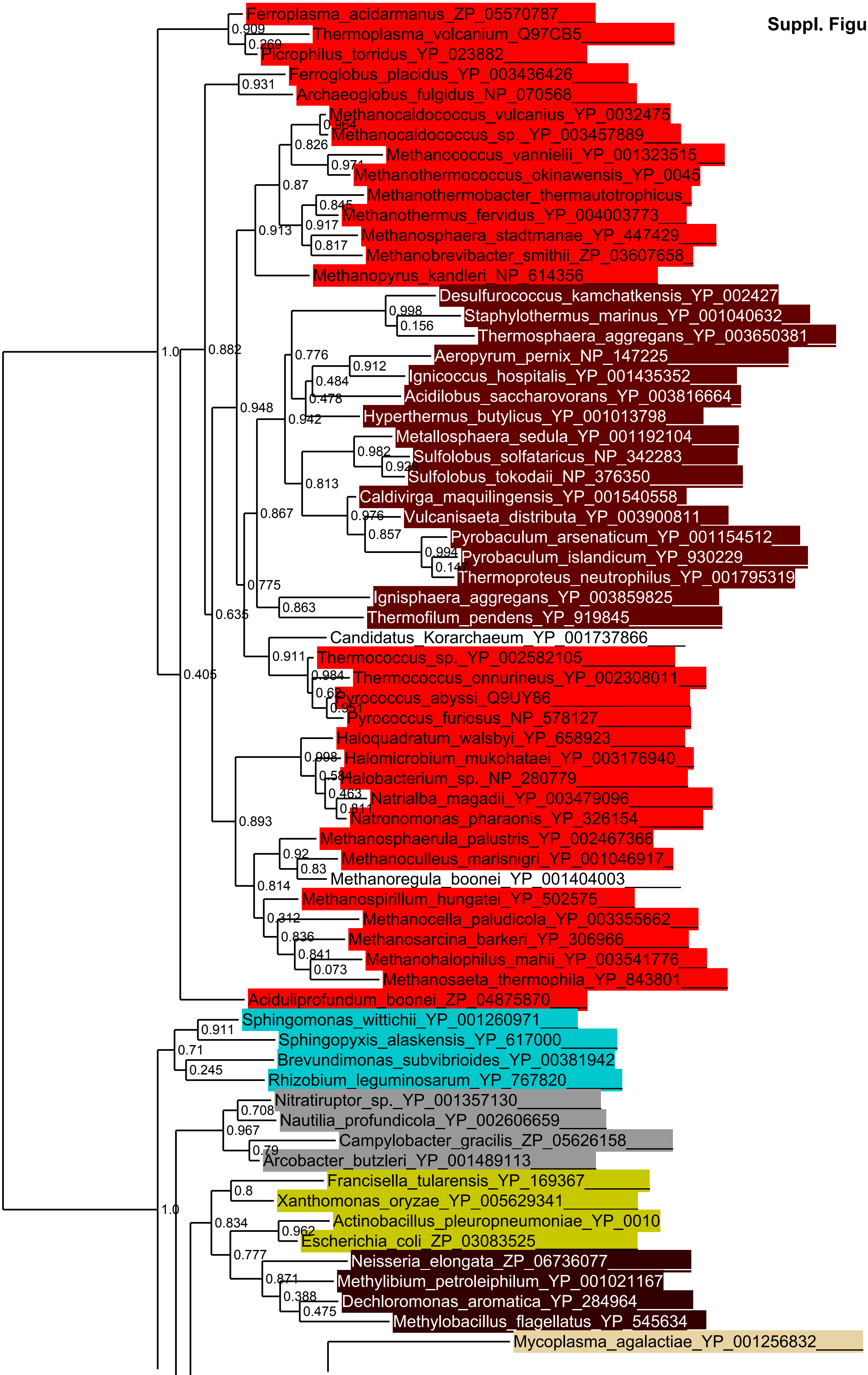

Suppl. Figure 8.

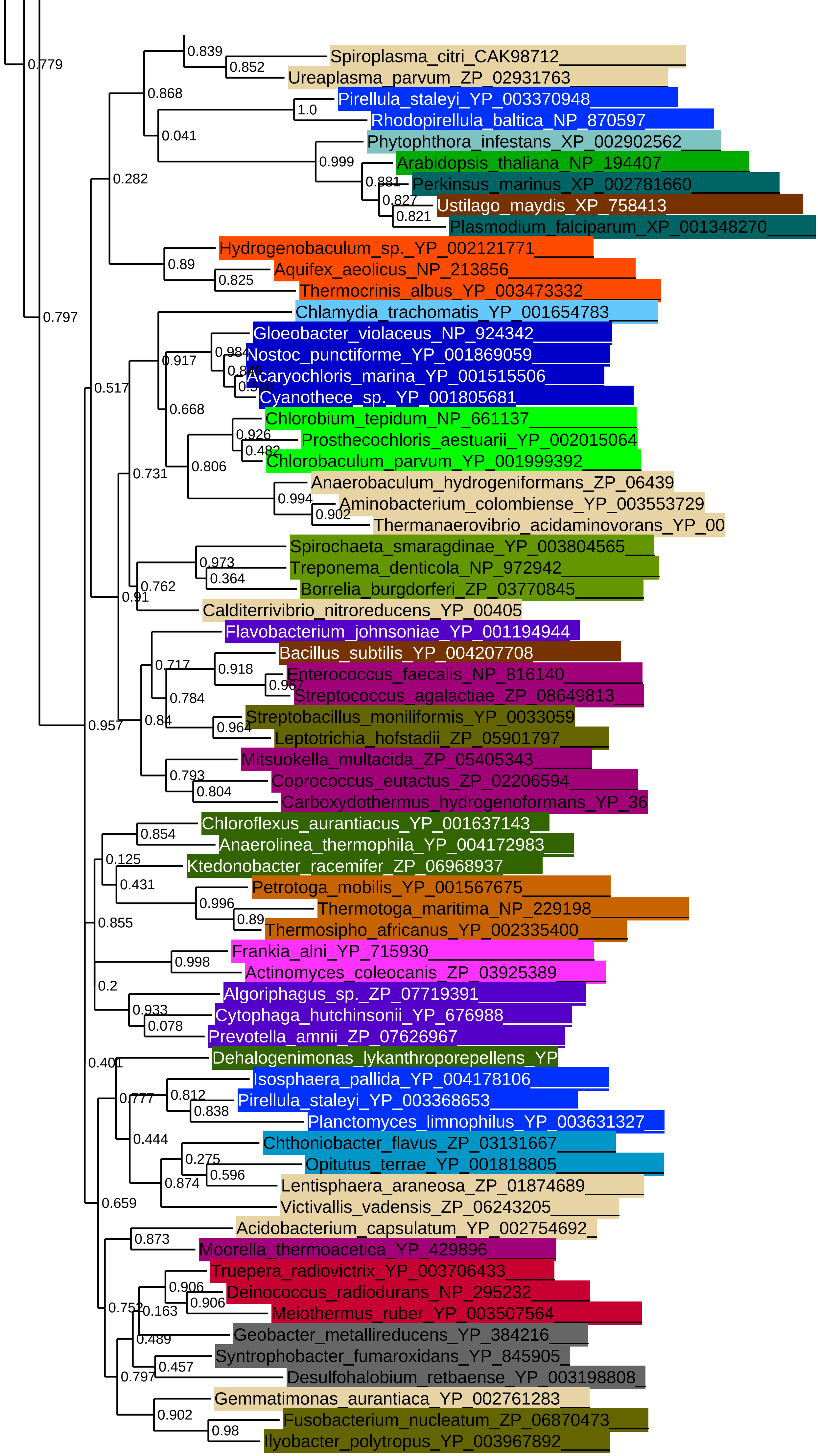
0.779
0.839
Spiroplasma_citri_CAK98712
0.852
Ureaplasma_parvum_ZP_02931763
0.868
Pirellula_staleyi_YP_003370948
1.0
Rhodopirellula_baltica_NP_870597
0.041
Phytophthora_infestans_XP_002902562
0.282
Arabidopsis_thaliana_NP_194407
0.999
Perkinsus_marinus_XP_002781660
0.881
Ustilago_maydis_XP_758413
0.827
0.821
Plasmodium_falciparum_XP_001348270
Hydrogenobaculum_sp._YP_002121771
0.89
Aquifex_aeolicus_NP_213856
0.825
Thermocrinis_albus_YP_003473332
0.797
Chlamydia_trachomatis_YP_001654783
Gloeobacter_violaceus_NP_924342
0.984
Nostoc_punctiforme_YP_001869059
0.917
0.849
Acaryochloris_marina_YP_001515506
0.517
0.518
Cyanothece_sp._YP_001805681
0.668
Chlorobium_tepidum_NP_661137
0.926
Prosthecochloris_aestuarii_YP_002015064
0.482
Chlorobaculum_parvum_YP_001999392
0.806
0.731
Anaerobaculum_hydrogeniformans_ZP_06439
0.994
Aminobacterium_colombiense_YP_003553729
0.902
Thermanaerovibrio_acidaminovorans_YP_00
Spirochaeta_smaragdinae_YP_003804565
0.973
Treponema_denticola_NP_972942
0.364
0.762
Borrelia_burgdorferi_ZP_03770845
0.91
Calditerrivibrio_nitroreducens_YP_00405
Flavobacterium_johnsoniae_YP_001194944
Bacillus_subtilis_YP_004207708
0.717
0.918
Enterococcus_faecalis_NP_816140
0.967
0.784
Streptococcus_agalactiae_ZP_08649813
Streptobacillus_moniliformis_YP_0033059
0.84
0.957
0.964
Leptotrichia_hofstadii_ZP_05901797
Mitsuokella_multacida_ZP_05405343
0.793
Coprococcus_eutactus_ZP_02206594
0.804
Carboxydothermus_hydrogenoformans_YP_36
Chloroflexus_aurantiacus_YP_001637143
0.854
Anaerolinea_thermophila_YP_004172983
0.125
Ktedonobacter_racemifer_ZP_06968937
0.431
Petrotoga_mobilis_YP_001567675
0.996
Thermotoga_maritima_NP_229198
0.855
0.89
Thermosipho_africanus_YP_002335400
Frankia_alni_YP_715930
0.998
Actinomyces_coleocanis_ZP_03925389
0.2
Algoriphagus_sp._ZP_07719391
0.933
Cytophaga_hutchinsonii_YP_676988
0.078
Prevotella_amnii_ZP_07626967
0.401
Dehalogenimonas_lykanthroporepellens_YP
Isosphaera_pallida_YP_004178106
0.777
0.812
Pirellula_staleyi_YP_003368653
0.838
Planctomyces_limnophilus_YP_003631327
0.444
Chthoniobacter_flavus_ZP_03131667
0.275
Opitutus_terrae_YP_001818805
0.596
0.874
Lentisphaera_araneosa_ZP_01874689
0.659
Victivallis_vadensis_ZP_06243205
Acidobacterium_capsulatum_YP_002754692
0.873
Moorella_thermoacetica_YP_429896
Truepera_radiovictrix_YP_003706433
0.906
Deinococcus_radiodurans_NP_295232
0.752
0.163
0.906
Meiothermus_ruber_YP_003507564
Geobacter_metallireducens_YP_384216
0.489
Syntrophobacter_fumaroxidans_YP_845905
0.457
0.797
Desulfohalobium_retbaense_YP_003198808
Gemmatimonas_aurantiaca_YP_002761283
0.902
Fusobacterium_nucleatum_ZP_06870473
0.98
Ilyobacter_polytropus_YP_003967892
